# An Updated and Comprehensive Review Exploring the Gut–Brain Axis in Neurodegenerative Disorders and Neurotraumas: Implications for Therapeutic Strategies

**DOI:** 10.3390/brainsci15060654

**Published:** 2025-06-18

**Authors:** Ahmed Hasan, Sarah Adriana Scuderi, Anna Paola Capra, Domenico Giosa, Andrea Bonomo, Alessio Ardizzone, Emanuela Esposito

**Affiliations:** 1 Department of Chemical, Biological, Pharmaceutical and Environmental Sciences, University of Messina, 98166 Messina, Italy; ahmed.hasan@unicam.it (A.H.); sascuderi@unime.it (S.A.S.); annapaola.capra@unime.it (A.P.C.); domenico.giosa@unime.it (D.G.); andrea.bonomo02@universitadipavia.it (A.B.); eesposito@unime.it (E.E.); 2 Center of Neuroscience, School of Advanced Studies, University of Camerino, 62032 Camerino, Italy; 3 Department of Mathematics, University of Pavia, 27100 Pavia, Italy

**Keywords:** gut–brain axis, neurodegenerative diseases (NDDs), neurotrauma, neuroinflammation, oxidative stress

## Abstract

The gut–brain axis (GBA) refers to the biochemical bidirectional communication between the central nervous system (CNS) and the gastrointestinal tract, linking brain and gut functions. It comprises a complex network of interactions involving the endocrine, immune, autonomic, and enteric nervous systems. The balance of this bidirectional pathway depends on the composition of the gut microbiome and its metabolites. While the causes of neurodegenerative diseases (NDDs) vary, the gut microbiome plays a crucial role in their development and prognosis. NDDs are often associated with an inflammation-related gut microbiome. However, restoring balance to the gut microbiome and reducing inflammation may have therapeutic benefits. In particular, introducing short-chain fatty acid-producing bacteria, key metabolites that support gut homeostasis, can help counteract the inflammatory microbiome. This strong pathological link between the gut and NDDs underscores the gut–brain axis (GBA) as a promising target for therapeutic intervention. This review, by scrutinizing the more recent original research articles published in PubMed (MEDLINE) database, emphasizes the emerging notion that GBA is an equally important pathological marker for neurological movement disorders, particularly in Alzheimer’s disease, Parkinson’s disease, multiple sclerosis, amyotrophic lateral sclerosis, Huntington’s disease and neurotraumatic disorders such as traumatic brain injury and spinal cord injury. Additionally, the GBA presents a promising therapeutic target for managing these diseases.

## 1. Introduction

Neurodegenerative diseases, such as Alzheimer’s disease (AD), Parkinson’s disease (PD), multiple sclerosis (MS), and amyotrophic lateral sclerosis (ALS), are among the most prominent and growing global health challenges, due to their steadily increasing prevalence and the lack of effective treatments capable of halting or reversing their progression. Despite scientific advances, available treatment options still focus solely on symptomatic relief, leaving a significant therapeutic gap. In recent years, the “gut–brain axis” (GBA) has emerged as a promising direction in neurological research. It refers to a bidirectional communication network linking the gastrointestinal tract and the central nervous system. This relationship is not limited to anatomical aspects but also encompasses complex neural, immune, and endocrine signals [1]. This complex interplay allows for continuous biochemical crosstalk between the gastrointestinal tract and the brain, with profound implications for both neurological health and disease. This axis consists of several key components, most notably the gut microbiome (beneficial bacteria in the gut), the intestinal barrier, the vagus nerve, and the immune system. These components interact through chemical, neurological, and immune signals that directly or indirectly impact brain health and function [2]. One of the most compelling aspects of this axis is the emerging recognition of the gut microbiota as a key regulatory component. The gut harbors trillions of microorganisms, bacteria, viruses, fungi, and archaea that contribute to host physiology by regulating digestion, metabolism, immune responses, and even behavior [3]. The microbial metabolites they produce, such as short-chain fatty acids (SCFAs), neurotransmitter precursors, and bile acids, are now known to influence brain function directly or indirectly through systemic and neural pathways [4]. In a healthy state, the microbiota and host systems maintain a delicate homeostasis; however, disruptions in this balance, known as dysbiosis, have been associated with a range of neurodegenerative and neurotraumatic conditions [5]. A growing body of evidence suggests that neurodegenerative diseases (NDDs), including AD, PD, MS, ALS, and Huntington’s disease (HD), are not merely diseases of the brain but may originate, or at least be influenced, by pathological changes in the gut [6]. Indeed, altered gut microbiome profiles have been observed in patients with these disorders, often accompanied by signs of chronic systemic inflammation, gut permeability (“leaky gut”), and immune dysregulation [7]. These findings challenge the classical view of NDDs as strictly central disorders and position the gut as a peripheral driver of CNS pathology. Moreover, neurotraumatic disorders, such as traumatic brain injury (TBI) and spinal cord injury (SCI), also exhibit gut-related alterations shortly after injury [8]. These changes include rapid shifts in microbial diversity, disruption of intestinal barrier function, and translocation of bacteria or endotoxins into the systemic circulation, all of which can exacerbate neuroinflammation and impede neural recovery [8]. As such, the gut microbiota may play an underappreciated role not only in the initiation and progression of neurodegenerative conditions but also in modulating recovery after acute neurological insults. Crucially, interventions aimed at modulating the gut microbiome, such as prebiotics, probiotics, dietary changes, fecal microbiota transplantation (FMT), and microbial metabolite-based therapies, have shown promise in preclinical models and, increasingly, in clinical studies [9]. Restoring a healthy microbial environment may reduce neuroinflammation, support neuronal survival, and improve cognitive or motor outcomes [9]. Notably, short-chain fatty acid-producing bacteria, such as certain species of *Lactobacillus* and *Bifidobacterium*, are thought to promote neuroprotection via anti-inflammatory and metabolic pathways [10].

This review aims to provide an updated and integrative perspective on the role of the gut–brain axis in neurodegenerative and neurotraumatic disorders, highlighting both the pathophysiological mechanisms and the emerging therapeutic opportunities in the more recent literature data published on PubMed (MEDLINE). We focus on the interplay between microbiota dysbiosis, immune activation, and neural degeneration, and explore how modulation of the GBA may offer new strategies to prevent or manage debilitating neurological conditions. Special emphasis was placed on translational models and human studies to bridge basic science findings with clinical application.

## 2. Search Strategy

The PubMed (MEDLINE) and Google Scholar bibliographic databases were used for the literature search. We considered original research articles of high quality that explored the role of the GBA in NDDs and neuro-traumas, with particular attention to potential therapeutic implications. The search covered the literature from database inception until 30 April 2025. We employed combinations of keywords related to the gut–brain axis, neurodegenerative diseases, and neuro-traumatic disorders. The specific search terms included the following:

Gut–brain Axis: gut–brain axis, gut microbiota, gut microbiome, microbiota–gut–brain axis, short-chain fatty acids, SCFA, intestinal permeability, dysbiosis.

Neurodegenerative Diseases: neurodegeneration, neurodegenerative disorders, Alzheimer’s disease, Parkinson’s disease, multiple sclerosis, amyotrophic lateral sclerosis, Huntington’s disease.

Neuro-traumas: traumatic brain injury, TBI, spinal cord injury, SCI, neurotrauma, CNS injury.

Therapeutic Approaches: probiotics, prebiotics, synbiotics, microbiome modulation, anti-inflammatory therapy, dietary intervention.

Only articles published in peer-reviewed journals and written in English were included. Studies were excluded if they were conference abstracts, editorials, letters, or reviews unless they provided relevant references to original research articles.

## 3. Molecular Mechanisms of GBA in Neuroinflammatory-Based Disorders

**Figure 1:**
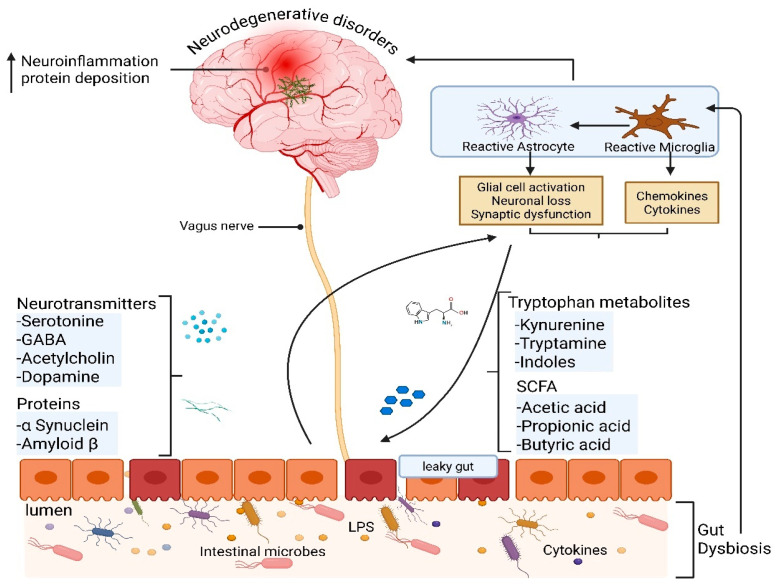
Schematic Representation of the GBA. The gut–brain axis is a bidirectional communication network connecting the central nervous system (CNS) and the gastrointestinal tract. This interplay involves neural (vagus nerve), endocrine (HPA axis), immune, and metabolic pathways. Gut microbiota plays a key role by modulating brain function through microbial metabolites (e.g., short-chain fatty acids), neurotransmitter precursors, and inflammatory signals. Alterations in gut barrier integrity and dysbiosis can impact brain health and are increasingly implicated in neurological and psychiatric disorders.

The GBA axis operates through several major molecular routes, including microbial metabolites, cytokine signaling, vagus nerve activation, and barrier integrity, each of which can influence neuroinflammation and neuronal survival, as illustrated in Figure 1.

A pivotal component of gut–brain communication lies in the production of microbial metabolites, which serve as systemic messengers capable of crossing the intestinal barrier and, in some cases, the blood–brain barrier (BBB) [11]. Among these, SCFAs such as acetate, propionate, and butyrate are of particular interest [12]. Produced primarily through the fermentation of dietary fibers by commensal bacteria, SCFAs exert a broad spectrum of effects on host physiology [12]. They have been shown to modulate the function of microglia, the resident immune cells of the CNS, by promoting anti-inflammatory phenotypes and attenuating proinflammatory cytokine release [13]. In models of multiple sclerosis and PD, butyrate supplementation has been linked to reduced disease severity and slowed progression, suggesting a neuroprotective role through immune modulation and epigenetic regulation of inflammatory genes [14]. Additionally, SCFAs regulate the permeability of the BBB by influencing tight junction protein expression, such as occludin and claudin-5 [15]. A disrupted BBB is a hallmark of many neuroinflammatory disorders and allows for the infiltration of peripheral immune cells into the CNS, further exacerbating neuroinflammation. Through their capacity to reinforce BBB integrity, SCFAs indirectly protect the brain from systemic immune activation.

Another metabolite class gaining attention is that of the tryptophan-derived indoles, which act on the aryl hydrocarbon receptor (AhR) expressed in both immune cells and astrocytes [16]. AhR activation can shift the inflammatory tone of the CNS toward a more homeostatic state, and microbial dysbiosis may impair this pathway by reducing the availability of indole ligands [16]. This mechanism has been implicated in neurodegeneration, particularly in MS, where AhR dysfunction contributes to demyelination and neuronal loss [16].

The gut microbiota exerts a profound influence on the systemic immune system, both through its antigenic components (e.g., lipopolysaccharides or LPS) and through the modulation of local and systemic cytokine profiles [17]. In conditions of dysbiosis, an increase in Gram-negative bacteria leads to elevated levels of LPS, which can translocate into the bloodstream due to increased intestinal permeability (“leaky gut”) [7]. Circulating LPS engages Toll-like receptor 4 (TLR4) on peripheral immune cells and brain-resident microglia, leading to the production of proinflammatory cytokines such as IL-1β, TNF-α, and IL-6 [18]. These cytokines can cross the BBB or affect the brain indirectly through endothelial activation and signaling cascades that prime microglial cells [18]. Chronic exposure to such inflammatory stimuli has been shown to contribute to neuronal dysfunction, synaptic loss, and the formation of protein aggregates, such as amyloid-beta plaques in AD and α -synuclein aggregates in PD [19]. Thus, gut-derived inflammatory signals can serve as upstream triggers for neuropathological cascades in neurodegenerative conditions. Additionally, the gut-associated lymphoid tissue (GALT) plays a crucial role in training and regulating T cell populations [20]. Dysbiosis-driven alterations in GALT output can result in skewed Th17/Treg balances, favoring a proinflammatory milieu that infiltrates the CNS [21]. In diseases like MS, Th17 cells are pathogenic and directly implicated in demyelination [22]. Altered microbial communities can increase the differentiation of Th17 cells via specific taxa, such as segmented filamentous bacteria, highlighting the causal role of gut microbes in shaping neuroinflammation [23].

The vagus nerve serves as a direct neural conduit between the gut and the brain and is a critical player in the GBA [24]. It contains both afferent and efferent fibers and can sense microbial-derived metabolites, hormones, and inflammatory mediators from the gut lumen [25]. Activation of vagal afferents influences brain regions involved in mood, cognition, and autonomic control, such as the hypothalamus and brainstem nuclei [26]. Emerging evidence suggests that certain probiotic strains can activate vagal pathways to exert anti-inflammatory and neurobehavioral effects. For instance, *Lactobacillus rhamnosus* has been shown to modulate GABA receptor expression in the brain via vagal signaling, altering anxiety-related behavior in mice [27]. Disruption of vagal transmission, either surgically or pharmacologically, abolishes these effects, underscoring the importance of this neuronal route in microbiota–brain communication. In the context of neuroinflammation, the cholinergic anti-inflammatory reflex, mediated through efferent vagal fibers, can inhibit the release of proinflammatory cytokines from peripheral macrophages [28]. Enhancing this pathway via microbiota modulation or vagal nerve stimulation could act as a potential therapeutic strategy in inflammatory and NDDs.

Both the intestinal epithelial barrier and the BBB operate as critical checkpoints in the GBA. Integrity of these barriers is essential in maintaining compartmentalization between the gut, systemic circulation, and CNS. In neurodegenerative disorders, increased permeability of these barriers is commonly observed and contributes to the pathological spread of inflammation. Microbial dysbiosis and the resulting metabolic changes can impair tight junction integrity in the gut epithelium, leading to enhanced translocation of bacteria and endotoxins [29]. This phenomenon not only fuels systemic inflammation but also has consequences for the BBB, where similar disruption facilitates the entry of peripheral immune cells and inflammatory mediators into the CNS [29]. Cytokines such as TNF-α and IL-1β, originating from the gut or peripheral sources, can further deteriorate BBB function by downregulating tight junction proteins and activating endothelial cells [30]. This bidirectional barrier breakdown, where gut leakiness influences BBB permeability and vice versa, creates a feedforward loop that perpetuates neuroinflammation and contributes to neuronal damage [31]. Targeting barrier integrity and neuroinflammation through dietary, pharmacological, or microbial interventions is, therefore, a promising avenue for halting disease progression [32,33].

## 4. NDDs

### 4.1. Pathophysiology of AD

AD is a progressive neurodegenerative disorder characterized by the extracellular deposition of Beta-amyloid plaques (Aβ) and the intracellular aggregation of tau neurofibrillary tangle (NFT) [34]. Beta-amyloid plaques are composed primarily of Aβ peptides, which are formed by the cleavage of the amyloid precursor protein (APP). These plaques interfere with communication between cells and can activate inflammatory responses, potentially leading to the death of neurons. NFTs arise from intracellular accumulations of hyperphosphorylated, microtubule-associated tau proteins. In the healthy brain, these tau proteins play an important role in stabilizing microtubules and axonal transport within nerve cells. In AD, hyperphosphorylation of tau proteins leads to their separation from microtubules. These tau proteins then aggregate into insoluble spiral filaments, which ultimately form NFTs. This process leads to loss of function and death of the affected nerve cells [35]. The combined effect of these pathological features results in brain atrophy and the cognitive decline observed in AD patients. Genetic factors, such as mutations in the *APP* gene and variations in the Apolipoprotein E (*APOE*) gene, have been implicated in increasing the risk of developing AD. Additionally, neuroinflammation and oxidative stress are believed to play significant roles in the disease’s progression [36]. The interaction of environmental influences throughout the entire lifespan, coupled with a possible genetic predisposition, is now considered a possible multifactorial cause. Intensive research has also shown that co-pathologies with enormous complexities of multiple pathological elements, such as hippocampal sclerosis, Lewy bodies, neuroinflammation, non-AD tauopathy, and cerebrovascular diseases, may also play a causal role in AD development [37].

Today’s technological advances enable the measurement of Aβ and tau in vivo, for example, through cerebrospinal fluid analysis or molecular imaging [38]. Studies have shown that these neuropathological changes in AD began decades before the first clinical symptoms [39].

#### Therapeutic Strategies Focusing on the GBA in AD

The new research focuses on understanding GBA activity, which has opened up a new therapeutic strategy for AD (Table 1). Many studies indicate that gut dysbiosis interferes with Alzheimer’s pathologies, such as beta-amyloid deposition, tau hyperphosphorylation, and neuroinflammation [40,41].

Dysbiosis could contribute to the pathogenesis of AD, generating a cascade of events. These include increased permeability of the intestinal barrier and activation of the immune system, causing neuroinflammation, and degeneration of brain cells and inflammation that disrupts the BBB, which may be partly due to dysregulated tight junctions due to decreased expression of tight junction proteins, particularly occludin and claudin-5 [42]. Some bacteria create and release amyloid peptides, LPS, and SCFA, which also activate inflammatory signals by releasing substances that enhance the pathological cascade of AD [43]. In a study conducted by Kozakhmetov et al., the results revealed a decrease in the Firmicutes/Bacteroidetes ratio, a decrease in the presence of beneficial bacteria such as Bifidobacterium, and an increase in pro-inflammatory cytokines such as IL-1α and IL-8. These changes were associated with changes in gut microbiota composition and serum adiponectin levels in AD patients [44]. Probiotic strains, particularly Lactobacillus and Bifidobacterium, have been shown to positively influence gut microbiota composition and cognitive function in AD patients through the production of neurotransmitters such as serotonin and gamma-aminobutyric acid (GABA) and reduction of neuroinflammation [45]. Prebiotics that promote the growth of beneficial gut microbes may aid in restoring gut balance, potentially reducing the deleterious effects of gut dysbiosis on neurodegeneration [46]. Together, this evidence supports the idea that microbiota contributes to the pathogenesis of AD. Fecal Microbiota Transplant has been used as a novel strategy in AD treatment. In animal studies, cognitive function was improved by reducing Aβ aggregation and modulating the inflammatory pathway. However, the clinical trial for this method is in the early stage. A diet rich in fiber, polyphenols, and omega-3 fatty acids, such as the Mediterranean or ketogenic diet, can modulate the gut microbiome in ways that support brain health. Moreover, polyphenols from berries, green tea, and curcumin have been shown to exert neuroprotective effects by reducing oxidative stress and inflammation along the GBA [47]. The immune system and its relationship with the gut microbiome are one of the axes that were studied. Researchers are putting forward the mechanisms linking the gut microbiome to AD that relate to the immune system. Some of the microbial metabolites affect inflammation in the gut and can even enter the bloodstream, where they can affect the immune system throughout the body. Gut microbial sulfonolipids (SoLs) have been shown to suppress inflammation by acting on Toll-like receptor 4 (TLR4), blocking LPS binding, and reducing macrophage polarization towards a pro-inflammatory state [48]. Changes in gut microbiota can influence systemic immune cell populations, such as increased CD4+ and CD8+ T cells, which correlate with neuroinflammatory markers in AD models [49]. Since chronic gut inflammation is a major driver of neuroinflammation in AD [50,51,52], therapeutic approaches targeting the gut-associated lymphoid tissue (GALT), including anti-inflammatory compounds and immune-modulating agents, are being investigated to reduce peripheral and central inflammation linked to AD pathology [53,54]. Apolipoprotein E4 (ApoE4) is the primary genetic risk factor for AD and acts primarily on lipid transport and maintenance of cholesterol balance. It is a protein involved in the metabolism of fats in the body, contributing to the development of amyloid plaque. ApoE4 elevates low-density lipoprotein (LDL) levels, raising the risk of cardiovascular disease and neurological diseases such as AD [55,56]. Future research should shed light on creating customized therapies based on personalized interventions that alter the gut microbiome, such as the use of probiotics, prebiotics, and dietary techniques that support brain health and improve the gut microbial balance. Intensive clinical research on FMT in AD patients should be conducted to better evaluate its safety, efficacy, and long-term effects. Moreover, it is critical to expand our knowledge of how immune system–gut interactions and microbial metabolites impact the development of AD, especially when considering genetic risk factors like apoE4. Combining microbiology, immunology, neuroscience, and genetics in a multidisciplinary approach will aid in the research of novel therapeutic and diagnostic strategies that support more accurate and efficient disease prevention and treatment.

**Table 1:** Overview of Studies Exploring the GBA in AD.

Intervention	Key Findings—Related Targets and Pathways	Model/Limitations	Effect on AD	Ref.
Compared germ-free mice to normal mice; tested effects of gut microbiota colonization and SCFA treatment on BBB permeability	Germ-free mice had leaky BBB with reduced tight junction proteins (occludin, claudin-5); gut microbiota and SCFAs restored BBB integrity	Mouse model/Short time intervention, Species differences between human and mouse model	Gut microbiota helps maintain BBB integrity; disruption may contribute to AD progression through increased brain vulnerability	[42]
Aanalyzing gut microbiota composition and blood cytokine levels in AD patients	AD patients exhibited a decreased Firmicutes/Bacteroidetes ratio, indicating gut dysbiosis. Elevated levels of proinflammatory cytokines, including IL-1β, IL-6, IL-8, and TNF-α, were detected in the blood of AD patients	Human subjects/Sample size, Does not prove the causality between dysbiosis and AD	Gut dysbiosis may contribute to systemic inflammation, which could exacerbate neuroinflammatory processes involved in AD pathogenesis	[44]
Gut microbial SoLs	SoLs bind to TLR4 and inhibit LPS binding. This action suppresses TLR4-mediated NF-κB signaling, leading to reduced production of pro-inflammatory cytokines (e.g., TNF-α, IL-6) and inhibition of macrophage M1 polarization and as a result neuroinflammation	Mouse model/Translational Gap, Complexity of Gut Microbiota, Limited Scope on Long-term Effects	Given the role of TLR4-mediated neuroinflammation in AD pathogenesis, SoLs’ ability to modulate TLR4 signaling suggests a potential protective effect against AD by reducing neuroinflammatory responses	[48]
Gut microbiota modulation and intervention with Huanglian Jiedu Decoction (HLJDD)	Altered SCFA and tryptophan-kynurenine metabolism; reduced pro-inflammatory cytokines and immune imbalance via gut–brain axis	Mouse Model/Limited Behavioral Analysis, short duration, Translation to Humans	Reduced neuroinflammation, decreased Aβ plaques, and improved cognitive function in AD model mice	[49]
Measurement of fecal calprotectin levels (a marker of intestinal inflammation)	Calprotectin levels increase with age, which was associated with more brain amyloid burden in AD patients	Human Cohort Study/The sample size, Biomarker Limitations, Potential confounders, No longitudinal data	Intestinal inflammation may contribute to amyloid accumulation and Alzheimer’s progression, but the study does not prove causality	[50,51,52]
Anvestigating GALT in 5xFAD transgenic mice, a model for AD	IL-17 protein production was reduced in gut-associated tissues (Peyer’s Patches and Mesenteric Lymph Nodes) of 5xFAD mice, despite normal Th17 cell numbers and IL-17 mRNA levels. In addition to downregulation of miR-155	5xFAD transgenic mouse model/focused only on IL-17, limited scope of immune factors, No longitudinal behavioral or cognitive correlation.	Impaired IL-17 production may weaken gut immune defense, promote dysbiosis, and worsen systemic and brain inflammation, potentially accelerating AD progression. Enhancing IL-17 signaling could be a therapeutic target	[53,54]
Analyzing the relation between APOE4 genotype, Aβ deposition, and episodic memory decline using data from the AD Research Initiative	APOE4 carriers exhibit a more rapid decline in episodic memory correlated with the duration of amyloid positivity, suggesting increased susceptibility to Aβ-related neurotoxicity	Human Cohort Study/Observational design, potential confounding factors, limit generalizability	The presence of the APOE4 allele accelerates cognitive decline in individuals with amyloid accumulation	[55,56]

### 4.2. Pathophysiology of PD

PD is a multifactorial and progressive neurodegenerative disorder characterized primarily by the degeneration of dopaminergic neurons in the substantia nigra pars compacta and the accumulation of misfolded α-synuclein protein in the form of Lewy bodies [57]. While traditionally framed as a brain-centric disease, growing evidence supports the notion that PD may originate, at least in part, outside the CNS, particularly in the gut. This emerging concept is grounded in both clinical and experimental observations and is shifting the understanding of PD pathogenesis toward a more systemic and integrative model. One of the most compelling pieces of evidence supporting this view is the early manifestation of non-motor symptoms such as constipation, which can precede classical motor signs by over a decade [58]. This has led to the formulation of the so-called “gut-first” hypothesis, wherein environmental triggers, potentially including pathogens, toxins, or dysbiotic microbial communities, may initiate pathological α-synuclein misfolding within the ENS [59]. From there, the abnormal protein could ascend to the CNS via the vagus nerve in a prion-like manner, a theory supported by both Braak’s staging model and animal studies demonstrating trans-synaptic propagation of α-synuclein from the gut to the brainstem [60]. In parallel, substantial alterations in gut microbiota composition have been documented in PD patients, with decreased abundance of beneficial, SCFA-producing taxa, such as Faecalibacterium and Roseburia [61], and an increase in potentially pro-inflammatory bacteria, including members of the Proteobacteria and Enterobacteriaceae families [62]. This dysbiotic environment is thought to compromise intestinal barrier integrity, facilitating the translocation of microbial components such as LPS into the systemic circulation. Once in circulation, these endotoxins can cross a permeabilized blood–brain barrier and activate immune receptors such as TLR4 on microglia, thereby triggering chronic neuroinflammation and promoting further α-synuclein aggregation [63]. Intriguingly, several genes associated with familial PD, including LRRK2, PINK1, and Parkin, intersect with immune and mitochondrial regulatory networks and are also expressed in peripheral tissues, including the gut [64]. LRRK2, in particular, has been linked to hyperinflammatory responses and is implicated in gastrointestinal disorders such as Crohn’s disease, suggesting a shared molecular vulnerability at the interface between the gut and the immune system [64]. The involvement of the GBA offers a unifying framework that connects these diverse elements and highlights the gut not merely as a passive participant but as a potential origin site and active driver of neurodegeneration. This paradigm opens new avenues for early detection and therapeutic intervention, emphasizing the need to consider systemic, microbiome-informed strategies in the management and prevention of PD.

#### Therapeutic Interventions Targeting GBA in PD

As stated, the GBA plays a significant role in PD, with increasing evidence suggesting that alterations in gut microbiota contribute to disease pathogenesis and progression. Recent studies have explored how gut microbiota, and associated metabolites affect PD through various mechanisms, revealing novel therapeutic opportunities (Table 2).

Liang et al. demonstrated that polystyrene nano-plastics accelerate α-syn aggregation in vitro and promote gut-to-brain propagation of the A53T mutant in vivo, resulting in motor deficits and increased oxidative stress, glial activation, and mitochondrial/lysosomal dysfunction, thus providing direct evidence of environmental nano-plastic exacerbation of PD pathology via the enteric route [65]. Ni and colleagues used 16S rRNA sequencing and transcriptomic analyses to show that fecal microbiota transplantation in MPTP-induced PD mice significantly restored motor function, increased striatal dopamine and serotonin levels, and elevated the expression of SCFA receptors (FFAR2 and FFAR3), pointing to a causal role of SCFAs, particularly acetate, propionate, and butyrate, in modulating neuroinflammation and neuronal survival [66]. Leem et al. explored pharmacological modulation of α7 nicotinic acetylcholine receptors (α7nAchR), showing that agonists GTS-21 and PNU-282987 reduce α-syn accumulation in both the brain and gut, promote autophagic clearance via AMPK-mTOR signaling, and exert anti-inflammatory effects [67]. These beneficial effects were abolished by the α7nAchR antagonist methyl-lycaconitine, supporting receptor-specific neuroprotective action [67]. Zhao et al., through a nested case-control design within the EPIC4PD cohort, identified 13 pre-diagnostic plasma microbial metabolites (including amino acids, indoles, bile acids, and hydroxy acids) nominally associated with PD risk, with pathway analysis implicating altered valine/leucine/isoleucine degradation and SCFA metabolism, especially in men, smokers, and overweight individuals [68]. Finally, Wu et al. established a gut-originated PD mouse model in which progressive α-syn spreading and motor dysfunction correlated with an increase in the genus Dubosiella, which disrupted BCAA metabolism, leading to systemic BCAA accumulation [69]. This metabolic disturbance impaired microglial lysosomal function and α-syn degradation. Notably, antibiotic-mediated depletion of gut microbiota reversed disease progression, highlighting a causal link between microbiota composition, metabolite accumulation, and PD pathology

Yu et al. introduced the Parkinson Gut Prediction Method (PGPM), a deep learning-based approach that accurately predicts PD using differential gut microbiota [70]. Their model, which showed high accuracy (0.85), represents a promising diagnostic tool that could aid in early PD detection by analyzing gut microbiota data [70]. This highlights the potential of gut microbiota as a diagnostic marker and the utility of advanced technologies like deep learning in PD research [70].

Wang and colleagues, in a recent article, investigated the role of *Akkermansia muciniphila* Akk11, a probiotic, in alleviating PD symptoms in a mouse model [71]. Akk11 administration improved behavioral deficits, reduced dopaminergic neuron loss, and mitigated microglial activation through inhibition of the NLRP3 inflammasome [71]. This study suggests that *A. muciniphila* supplementation could be a therapeutic strategy via the modulation of gut microbiota and its inflammatory responses.

Qi et al., instead, focused on *Lactiplantibacillus plantarum* SG5, a probiotic that demonstrated neuroprotective effects in MPTP-induced PD mice [72]. Their findings showed that SG5 modulated gut microbiota composition, reduced neuroinflammation, and improved motor deficits [72]. Importantly, SG5 acted through the GLP-1/PGC-1α signaling pathway, which plays a crucial role in neuroprotection and mitochondrial function, offering another promising probiotic-based therapeutic approach for PD [72].

In a different approach, Gao et al. studied the effects of a Brazilin-rich extract from *Caesalpinia sappan* L. in PD mice [73]. The extract enhanced gut microbiota composition, increased SCFA production, and reduced inflammation, which helped to alleviate PD-related motor deficits and neurodegeneration [73]. This underscores the potential of natural compounds in regulating the GBA, and consequently, in treating PD.

Qiao et al. explored the effects of inhibiting indoleamine 2,3-dioxygenase 1 (IDO-1), an enzyme involved in tryptophan metabolism, in PD [74]. Their study found that IDO-1 inhibition improved motor function, reduced inflammation, and promoted neurogenesis, with the therapeutic effects being dependent on gut microbiota [74]. These results advise that targeting IDO-1 could be a promising strategy for PD treatment by modulating both the gut microbiota and neuroinflammation.

Other therapeutic interventions targeting gut microbiota, including probiotics, natural compounds, and metabolic pathways, show promise in alleviating PD symptoms and potentially slowing disease progression. For instance, Shao et al. demonstrated the neuroprotective effects of *Enterococcus faecalis* (EF) in a mouse model of PD, showing that treatment with a non-pathogenic strain of EF (EF ATCC19433) alleviated dopaminergic neuronal damage and gastrointestinal dysfunction through the vagus nerve [75]. This study further supports the idea that gut microbes can influence brain function via the vagal pathway. Additionally, Shang et al. discovered that FLZ, a neuroprotective agent, improved the condition of PD mice by regulating the production of bile acids derived from microbiota, particularly glycourso-deoxycholic acid, which promotes dopaminergic neuron protection through the Nrf2 pathway [76]. Additionally, the probiotic approach was explored by Hassan et al., who combined Vinpocetine and *Lactobacillus* in rotenone-induced PD rats [77]. They observed a reduction in neuroinflammation, improved dopamine synthesis, and a regression of Lewy body inclusions [77]. Other studies, such as that by Chtioui et al., recommend that an active lifestyle could reduce intestinal inflammation and improve SCFA production in PD patients, confirming the benefits of a balanced diet and physical activity in managing the disease [78].

Bolen et al. focused on the connection between peripheral immune cells and gastrointestinal inflammation in PD, noting that iron dysregulation and mitochondrial dysfunction in immune cells could be pivotal in the pathogenesis of the disease [79]. Their research found shared immuno-metabolic features between PD and inflammatory bowel disease (IBD), suggesting that chronic inflammation in the gut might contribute to PD risk [79]. Interestingly, non-steroidal anti-inflammatory drugs (NSAIDs) were shown to modulate immune responses in inflamed individuals, providing a potential therapeutic avenue.

In a related study, Zaman et al. explored the effects of rotenone, a known mitochondrial toxin, on metabolic peptides in rats, highlighting how exposure to this toxin precedes hyperinsulinemia and inflammation, both of which are common in PD [80]. The study found that rotenone disrupted metabolic homeostasis, leading to significant changes in hormones like insulin, leptin, and glucagon-like peptide-1 (GLP-1) [80]. This dysregulation of metabolic peptides was linked to increased body weight and immune activation, pointing to a possible role of metabolic dysfunction and GBA disturbance in PD progression. In the future, focus should be on developing techniques that target the modulation of the composition of the gut microbiome to improve PD symptoms and delay its progression. A deeper study is also needed into the role of microbial metabolites and their impact on neuroinflammation and neuronal function. Large-scale clinical trials are also essential to evaluate the efficacy of probiotic and natural compound-based therapies. Furthermore, artificial intelligence techniques can be leveraged to improve early diagnosis of the disease through microbiome data analysis. Strategies to integrate a healthy lifestyle and diet to support gut–brain health should also be explored. Finally, studying the interaction between the immune system and the microbiome may open new avenues for innovative treatments for PD.

**Table 2:** Studies evaluating GBA in PD.

Intervention	Key Findings—Related Targets and Pathways	Model/Limitations	Effect on PD	Ref.
Effect of FMT from healthy donors into MPTP-induced PD mouse models. and the action of SCFAs through their receptors, FFAR2 and FFAR3, in modulating CNS functions	FMT increased gut levels of SCFAs in PD mice, upregulated SCFA receptors FFAR2 and FFAR3 in the CNS, enhanced dopamine and serotonin levels in the striatum, promoted the survival of dopaminergic neurons in the substantia nigra, and reduced glial cell activation	Mouse model/Most evidence is correlational not causality, limit generalizability, Species-specific microbiota differences, No human validation	FMT restored SCFA levels, improved dopaminergic neuron survival, reduced neuroinflammation, and increased dopamine and serotonin levels in the brain, leading to improved motor and neurological functions in PD mice	[66]
Administration of α7nAChR agonists, GTS-21 and PNU-282987 to subacute MPTP-induced PD mouse models	Treatment reduced α-synuclein accumulation in the brain and colon, activated AMPK-mTOR–mediated autophagy, promoting autophagic clearance of α-synuclein, suppressed pro-inflammatory mediators such as iNOS, IL-6, and TNF-α, in both brain and gut tissues, enhanced dopaminergic signaling and motor function, and exerted effects via α7nAChR, as confirmed by MLA inhibition	Mouse Model/Short duration, Mechanism not fully clear, Limited behavioral data, Focus on α-synuclein only	Activation of α7nAChRs mitigated α-synuclein pathology, reduced neuroinflammation, and preserved dopaminergic neurons, leading to improved motor function in PD mouse models	[67]
Prospective metabolome-wide association study (MWAS) analyzing pre-diagnostic plasma samples from the EPIC4PD cohort to identify microbial metabolites associated with future PD risk	Identified 13 microbial metabolites associated with PD risk, including amino acids, bile acids, indoles, and hydroxy acids, with Pathway analyses highlighting branched-chain amino acid degradation, such as valine, leucine, and isoleucine and SCFA metabolism, and stronger associations observed in men, smokers, and individuals with obesity	Human Cohort Study/Observational design only, limited population diversity, No causal inference	Alterations in gut microbial metabolites may serve as early biomarkers for PD risk, highlighting the potential role of the gut–brain axis in PD pathogenesis and offering avenues for early intervention strategies	[68]
Utilization of a gut-originated PD mouse model to investigate the role of gut microbiota in the propagation of pathological α-syn from the gut to the brain	Gut microbiota promoted the vagus nerve-mediated spread of pathological α-synuclein from the gut to the brain, triggered neuroinflammation through microglial activation and cytokine release, and worsened motor deficits compared to germ-free mice	Mouse Model/No human validation, short duration of study, Microbiota complexity underestimated	The study demonstrates that gut microbiota plays a crucial role in promoting the propagation of α-syn pathology from the gut to the brain, thereby exacerbating neuroinflammation and motor deficits characteristic of PD	[69]
A deep learning model used gut microbiota data and combines Combined Ranking using Random Forest Scores (CRFS) for feature selection, LSTM-penultimate to SVM Input Method (LSIM) for classification, and soft voting for prediction, based on data from 39 PD patients and their healthy spouses	PGPM demonstrated high predictive accuracy (85%) and AUC (0.92) in distinguishing PD patients from controls, with CRFS identifying key microbial features and the LSIM architecture enhancing classification through combined LSTM and SVM modeling	Computational Model with Human Microbiome Data/No experimental validation, Correlational model only, Potential overfitting risk, Lacks biological mechanisms	The study demonstrates that analyzing gut microbiota using advanced deep learning techniques can effectively predict Parkinson’s disease, highlighting the potential of gut microbiota as a non-invasive biomarker for early diagnosis	[70]
Oral supplementation of Akkermansia muciniphila strain Akk11 in mice induced with PD using MPTP	Akk11 suppressed activation of the NLRP3 inflammasome in microglia, reducing neuroinflammation, preserved dopaminergic neurons in the substantia nigra, improved MPTP-induced motor deficits, and protected colonic integrity	Mouse Model/Animal Model limitation, Short Duration of Treatment, Limited Sample Size, Focus on Specific Outcomes	Akk11 supplementation mitigated neurodegeneration and motor dysfunction in PD models by modulating neuroinflammatory pathways and enhancing gut health.	[71]
Oral administration of Lactiplantibacillus plantarum SG5 to mice induced with PD using MPTP	SG5 improved motor function and dopaminergic neuron survival in the substantia nigra, reduced α-synuclein levels, suppressed overactivation of microglia and astrocytes, restored BBB and gut barrier integrity, modulated gut microbiota, and activated the GLP-1/PGC-1α pathway effects, which was validated by pathway-specific inhibitors	Mouse Mode/Animal Model Limitations, short duration of treatment, Potential Confounding Factors	SG5 supplementation mitigated neurodegeneration and motor dysfunction in PD models by modulating neuroinflammatory pathways, enhancing gut health, and activating the GLP-1/PGC-1α signaling pathway	[72]
Oral administration of a 91.23% brazilin-enriched extract from Caesalpinia sappan L. (SE) to MPTP/p-induced PD mice	SE ameliorated motor deficits and improved dopaminergic neuron survival, reduced oxidative stress and neuroinflammation in the brain, restored gut microbiota balance by increasing Firmicutes and decreasing Bacteroidetes, increased SCFA (especially butyric acid) levels, enhanced intestinal barrier integrity via upregulation of ZO-1 and occludin, and reduced LPS leakage and systemic inflammation	Mouse Model/Animal Model Limitations, short duration of treatment, lack of Control Comparisons	SE mitigated neurodegeneration and motor dysfunction in PD models by modulating the gut–brain axis, reducing neuroinflammation, and restoring intestinal barrier integrity	[73]
Administration of 1-methyl-tryptophan (1-MT), an IDO-1 inhibitor, to MPTP-induced PD mice	IDO-1 inhibition improved motor behavior and dopaminergic neuron survival in the substantia nigra, reduced serum quinolinic acid and aryl hydrocarbon receptor levels in the striatum and colon, suppressed TLR4/NF-κB mediated neuroinflammation, leading to decreased iNOS and COX2 expression, promoted hippocampal neurogenesis indicated by increased DCX+ and SOX2+ cells, likely via activation of the BDNF/TrkB pathway, normalized SCFA levels, and required gut microbiota for its neuroprotective effects	Mouse Model/Animal Model Limitations, short duration of treatment, Translational relevance uncertain, Gut microbiota variability	IDO-1 inhibition mitigated neurodegeneration and motor dysfunction in PD models by modulating neuroinflammatory pathways, promoting neurogenesis, and regulating gut microbiota composition	[74]
Oral administration of Enterococcus faecalis (EF) to MPTP-induced PD mice	EF treatment alleviated neurofunctional impairments and gastrointestinal disorders associated with PD in mice, reversed dysbiosis of PD-related microbial communities induced by MPTP, and exerted its neuroprotective effects via the vagus nerve, as shown by diminished benefits following vagotomy	Mouse Model/Animal Model Limitations, short duration of treatment, Mouse-specific neural pathways, Vagus mechanism uncertain	EF supplementation mitigated neurodegeneration and motor dysfunction in PD models by modulating neuroinflammatory pathways and enhancing gut health through vagus nerve-mediated mechanisms	[75]
Administration of FLZ, a neuroprotective agent, to MPTP-induced PD mice	FLZ modulated gut microbiota, by downregulating *Clostridium innocuum*, increased glycoursodeoxycholic acid levels through enhanced bile salt hydrolase (BSH) enzymes activity, activated the Nrf2 antioxidant pathway, reduced neuroinflammation, protected dopaminergic neurons in the substantia nigra, and exerted its effects via the microbiota-GBA	Mouse Model/Animal Model Limitations, short duration of treatment, Single bacterial target	FLZ mitigated neurodegeneration and motor dysfunction in PD models by modulating gut microbiota composition, enhancing beneficial metabolites like GUDCA, and activating neuroprotective pathways	[76]
Co-administration of Vinpocetine and Lactobacillus in rotenone-induced PD rat model	Treatment with Vinpocetine and Lactobacillus increased tyrosine hydroxylase (TH) expression to restore dopamine synthesis, reduced oxidative stress (lower MDA, higher GSH), suppressed pro-inflammatory cytokines (IL-1β, TNF-α) and nitrite levels, and decreased accumulation of α-synuclein and tau proteins	Mouse Model/Animal model limitations, short duration of treatment, Absence of Mechanistic Insights	Ameliorated dopaminergic neurodegeneration, improved motor function, reduced inflammation and oxidative stress, and inhibited Lewy body formation in PD models	[77]
Engagement in an active lifestyle characterized by regular physical activity in individuals with PD	Physically active individuals showed increased SCFA production, reduced intestinal inflammation (lower fecal calprotectin), improved constipation symptoms, and potential beneficial modulation of the gut–brain axis in PD patients	preliminary Human Observational Study/Small Sample Size, Lack of Control for Physical Activity Types, No Evidence of Sex-Specific Differences, No microbiota analysis	The active lifestyle may alleviate gastrointestinal symptoms and reduce intestinal inflammation in PD patients, potentially contributing to improved overall health and quality of life	[78]
An analysis of peripheral blood mononuclear cells (PBMCs) from individuals with PD or IBD, focusing on iron metabolism and the influence of NSAIDs	PBMCs from PD and IBD patients show impaired iron storage and transport, leading to mitochondrial dysfunction characterized by dysmorphic mitochondria, suggesting a link to ferroptosis, with shared inflammatory and metabolic signatures; NSAID uses may modulate these transcriptional abnormalities	Human Clinical Sample Analysis/Sample size and selection, Cross-sectional design, NSAID effects variable, No microbiota profiling	The study suggests that iron dysregulation and mitochondrial dysfunction in peripheral immune cells may contribute to PD pathogenesis. NSAID-mediated modulation of these pathways indicates potential therapeutic avenues for PD	[79]
Administration of rotenone (a mitochondrial complex I inhibitor) to rats to model PD	Rotenone exposure disrupted metabolic homeostasis by altering peptide levels (decreased GLP-1, C-peptide, amylin; increased insulin, leptin, pancreatic polypeptide, peptide YY, gastric inhibitory polypeptide), induced systemic inflammation via TNF-α–expressing CD4+ T cells, and increased body weight, linking mitochondrial dysfunction to metabolic and immune disturbances	Rat Model/Species differences between rats and humans, Focus on short-term effects, No behavioral assessments	The study suggests that rotenone-induced mitochondrial dysfunction leads to metabolic peptide dysregulation and systemic inflammation, which may contribute to the pathogenesis of PD	[80]

### 4.3. Etiopathogenesis of MS

MS is an inflammatory, demyelinating autoimmune and neurodegenerative disease marked by immune cell infiltration, demyelination, axonal degeneration, and neuronal cell death in CNS [81]. It is generally considered to be an immune-mediated disease that occurs in genetically predisposed individuals [82]. The pathogenesis of MS is not yet fully understood. The development of MS is influenced by a complex interaction of genetic predisposition, environmental factors, and immune system dysregulation [83]. Focal immune cell infiltration is considered to be the initial cause of damage in MS [84].

Many studies suggest that viral, bacterial and fungal antigens, which are similar to autoantigens, cause an interaction between antigen-presenting cells and T lymphocytes. This stimulates T helper cell intervention and the adaptive immune response and thus plays an important role in the initiation and progression of MS [85]. MS is presumably predominantly T cell-controlled and is directed primarily against myelin sheath proteins produced by oligodendrocytes [86]. The resulting demyelination impairs rapid saltatory conduction of excitation and leads to clinical symptoms [85].

Antigen-presenting cells internalize antigens and initiate the production of specific cytokines (interleukin-12, -23, -4). These cytokines manage the differentiation of CD4⁺ T cells into distinct T helper (Th) cell cells (Th-1, -2 or Th-17 phenotypes), which in turn secrete specific cytokines [87]. Activated CD4⁺ T cells that recognize self-antigens are believed to attach to the luminal surface of endothelial cells of venules of the CNS. Upon the disruption of the blood–brain barrier, these T cells migrate into the brain, potentially contributing to neuroinflammatory processes [88].

Environmental factors have a greater influence on the onset of the disease, with Epstein–Barr virus (EBV) being the most important viral trigger [89]. Almost all MS patients have had previous exposure to EBV, and molecular mimicry between Epstein–Barr virus proteins and myelin antigens may trigger an autoimmune attack [90]. Studies have also been conducted on other viruses, such as human herpesvirus 6 (HHV-6) and cytomegalovirus (CMV), for their possible role in initiating the disease [91]. Additionally, vitamin D deficiency is closely linked to MS, as low vitamin D levels impair immune regulation by reducing anti-inflammatory responses and regulatory T cell function, making individuals more susceptible to autoimmune diseases [92]. Geographic studies show a higher prevalence of MS in populations residing at higher latitudes with lower exposure to sunlight. Lifestyle factors such as smoking contribute to the risk of the disease by promoting oxidative stress, enhancing inflammation, and accelerating neurodegeneration, leading to a more severe disease course [93]. Furthermore, emerging research suggests that gut microbiota imbalance plays a role in MS by disrupting the balance between pro-inflammatory and anti-inflammatory immune responses, which may influence disease progression [94].

#### Evaluating Gut–Brain-Based Treatments in MS

Recent studies indicate that immune activation in MS may begin in the gut (Table 3), and alterations in the immune response can lead to alterations in the gut microbiota [95]. It is well known that the brain controls the motor, sensory, and secretory activities of the digestive tract. Conversely, the intestine and its microbiota act on the brain and interact with behaviors particularly dietary, cognitive functions and mood stress [96,97]. Gut dysbiosis correlates with an increased level of bacterial lipopolysaccharides (LPSs) and related proteins such as LBP (LPS-binding protein) in CNS. LPSs act as exotoxins, activating B cells, stimulating cytokine production, and increasing oxidative stress [98].

To restore the balance of intestinal microbiota, Probiotics, when administered, can have beneficial effects on health and can be used as a treatment for intestinal problems [99]. Studies conducted in mice are contradictory. In some cases, the administration of probiotics had no effect; in other cases it was accompanied by an improvement in the pathology [99]. It is known that the Western diet, which is high in salt, saturated fats, protein, and sugar, is linked to an increased frequency of autoimmune diseases. Conversely, Mediterranean diets, with low-calorie intake, rich in fruits, vegetables, and fish, lead to less inflammation and can have a favorable effect on the microbiota [100].

A fecal microbiota transplant consists of administering a new microbiota to a patient via feces from a healthy person, who is assumed to have a balanced microbiota. A few transplants of this type have been performed worldwide in patients with multiple sclerosis. The reported effects are in favor of an improvement in certain symptoms, particularly motor function and reduced disability scores, enhancements in gut microbiota composition, and reduction in inflammatory markers like IL-6 and TNF-α [101,102].

Several active compounds of gut microbiota belonging to various chemical classes have been studied in the past decade in relation to MS. Tryptamine, a byproduct of tryptophan produced by gut bacteria, reduces inflammation by altering gut microbiota and increasing butyrate levels [103]. Its derivative, 5-hydroxytryptamine (5-HT), regulates the central nervous system and improves mood, and its deficiency in MS patients often leads to depression. Serotonin regulators such as SSRIs are possible treatment options, which act by reducing microglial activation, oxidative stress, and neuroinflammation [104].

Another study illustrated that N-acetyl-5-methoxytryptamine (melatonin), which is a tryptamine derivative, shows promising effectiveness in the management of MS. Melatonin acts as a signaling molecule in the central nervous system, influencing immune responses, oxidative stress, and cell death [105]. It was indicated that the interaction between melatonin and the gut microbiota may trigger oxidative stress and inflammation [105]. Its deficiency has been linked to fatigue and depression associated with MS. Melatonin enhances neuronal antioxidant defenses by raising levels of CAT, SOD, GPx, and GSH, while microbial-derived antioxidants reduce inflammation via the Nrf2-ROS/NLRP3/IL1β pathway [105,106].

Given the sensitivity of serotonin and melatonin to light exposure, there may be a link between environmental factors, gut microbiota, and their deficiency in MS. SCFAs, such as acetate, butyrate, and propionate, produced by the gut microbiota, significantly influence immune responses, the balance of demyelination, remyelination, and oxidative stress [107]. Unlike SCFAs, long-chain fatty acids (LCFAs) can induce a pro-inflammatory state by activating Th17 cells [108,109]. In contrast, SCFAs exert anti-inflammatory effects by promoting cytokine production and restoring BBB integrity. The gut microbiota is critical in maintaining the balance between pro- and anti-inflammatory mediators. Additionally, SCFAs can modulate redox signaling through the Nrf2 pathway [108].

Probestel et al. demonstrated that some B cells build a kind of connection between gut bacteria and inflammatory sites in CNS and have an anti-inflammatory effect. It was suggested that gut microbiota-specific IgA+ B cells can act as systemic mediators in MS. This opens the possibility of using IgA as a biomarker of the disease and highlights the potential of IgA-producing cells as a therapeutic target during active neuroinflammation [110].

Future directions in MS research include focusing on the role of trained and innate immunity and gut health in disease progression, examining the impact of changes in the gut microbiome on the immune system’s response. Research is also moving toward the use of probiotics to improve microbiome balance and its impact on symptoms, as well as studying the effect of diets, such as the Mediterranean diet, on reducing inflammation and improving gut health. There is growing interest in therapies that target active compounds produced by microbiomes, such as tryptamine, serotonin, and melatonin, which influence inflammation and oxidative stress. The role of short-chain fatty acids in modulating the immune response and protecting the brain is also being explored. Finally, IgA-producing B cells are considered as a potential therapeutic target and biomarker for MS, opening new avenues for targeted immunotherapy.

**Table 3:** Articles assessing GBA in MS.

Intervention	Key Findings—Related Targets and Pathways	Model/Limitations	Effect on MS	Ref.
Measurement of fecal lipocalin-2 (Lcn-2) levels in patients with relapsing-remitting multiple sclerosis (RRMS) and in experimental autoimmune encephalomyelitis (EAE) mouse models	RRMS patients showed elevated fecal Lcn-2 levels, which correlated with reduced microbial diversity and increased intestinal inflammation, findings that were mirrored in EAE mouse models, supporting a link between gut dysbiosis and inflammation.	humanized transgenic mouse model/Small sample size, Heterogeneity of Microbiome, Focus on Specific Biomarkers, Short-term Observations, Limited clinical correlation	Fecal Lcn-2 serves as a sensitive, non-invasive biomarker for detecting gut dysbiosis and intestinal inflammation in MS. This highlights the role of gut health in MS pathogenesis and suggests potential for monitoring disease progression or response to therapies targeting the gut microbiome	[95]
Implementation of a Mediterranean diet in patients with MS.	Adherence to the Mediterranean diet improved physical and mental health-related quality of life measures, reduced disability levels, and likely mitigated MS symptoms through its anti-inflammatory effects	Human Clinical Study/Small sample size, Single healthcare setting, No control group, Lack of biochemical parameters, Self-reported dietary data	The Mediterranean diet appears to positively influence disease outcomes in MS patients by improving quality of life and reducing disability, potentially through its anti-inflammatory effects	[100]
Monthly FMT administered to patients with relapsing-remitting multiple sclerosis (RRMS) over a six-month period	FMT was safe and well-tolerated in RRMS patients, led to improved intestinal permeability in some cases, induced beneficial donor-specific shifts in gut microbiota, but showed no significant changes in Expanded Disability Status Scale scores or MRI outcomes	Human Randomized controlled trial (pilot RCT)/Small sample size, Lack of control group, Short-term follow-up, Limited efficacy data	The study suggests that FMT may improve gut-related parameters in MS patients, such as intestinal permeability and microbiota composition, without adverse effects. However, due to the small sample size and early termination of the study, further research is needed to assess its impact on neurological outcomes	[101,102]
Administration of tryptamine, a naturally occurring monoamine derived from tryptophan, in mouse models of EAE, which is commonly used to study MS	Tryptamine activates the aryl hydrocarbon receptor (AHR), suppresses neuroinflammation and paralysis in EAE models via AHR-dependent pathways, tryptamine effect was absent in mice lacking AHR in T cells, and modulates gut microbiota to increase anti-inflammatory butyrate production	Murine model/Not directly translating to human data, Animal model constraints	The study suggests that tryptamine, through AHR activation, can modulate immune responses and gut microbiota composition, leading to reduced neuroinflammation and amelioration of MS-like symptoms in animal models. This points to the potential of targeting the AHR pathway as a therapeutic strategy in MS	[103]
Treatment of RAW264.7 macrophage cells with microbe-derived antioxidants (MAs) to assess their effect on LPS-induced inflammation	Microbial antioxidants (MAs) activate the Nrf2 pathway, enhancing antioxidant enzyme expression and reducing ROS, while inhibiting NLRP3 inflammasome activation and associated cytokine release, with their anti-inflammatory effects dependent on Nrf2 signaling	In Vitro RAW264.7 cells/Cell line specificity, Lack of in vivo validation, Simplified inflammatory model, Translational relevance uncertain	MAs attenuate LPS-induced inflammatory responses by activating the Nrf2 pathway, which in turn inhibits the ROS/NLRP3/IL-1β signaling axis. This mechanism suggests potential therapeutic applications of MAs in managing inflammation-related diseases	[105,106]
Investigation of microglial development and function in germ-free (GF) mice, mice with limited microbiota complexity, and mice recolonized with complex microbiota	Germ-free (GF) mice show impaired microglial maturation and function due to microbiota absence, with restoration partially achieved through complex microbiota or SCFA supplementation, highlighting SCFAs and FFAR2 signaling as key regulators of microglial homeostasis	Mouse Model/Mouse model constraints, Translational relevance uncertain, Microbiota-microglia mechanism unclear	The study demonstrates that host microbiota is vital for the proper maturation and function of microglia, suggesting that disruptions in gut microbiota could impact CNS immunity and potentially contribute to neurological disorders	[107]
Administration of butyrate, a microbial fermentation product, to assess its effect on the differentiation of colonic Treg cells in mice	Butyrate promotes colonic Treg cell differentiation by enhancing histone H3 acetylation at the Foxp3 locus and ameliorates colitis in a CD4⁺ CD45RB^high^ T cell transfer model in Rag1⁻/⁻ mice, highlighting its epigenetic and immunomodulatory effects	Mouse Model and in vitro cell assays/small sample sizeLimited cell types analyzed, Model limitations	The study demonstrates that butyrate, produced by commensal microbes, plays a crucial role in maintaining intestinal immune homeostasis by promoting the differentiation of colonic Treg cells, thereby suppressing inflammatory responses	[108,109]
Investigation of gut microbiota-specific IgA⁺ B cells in CNS of individuals with active MS	IgA⁺ B cells specific to gut microbiota were found in the CNS during active MS, indicating that gut-derived immune responses can mediate CNS inflammation and may serve as biomarkers or therapeutic targets	Human Subjects and mouse model/Human sample variability, Cross-sectional design, small sample size and Diversity	The study indicates that gut microbiota-specific IgA⁺ B cells contribute to CNS inflammation in active MS, highlighting a novel gut–brain immune axis that may be pivotal in disease progression	[110]

### 4.4. Pathophysiology of ALS

ALS is a rare neurodegenerative disease that irreversibly affects the central nervous system [111]. Its main characteristic is the loss of motor neurons and atrophy of the respiratory muscles, which leads to patient death [112]. The disease progressively causes the degeneration and eventual death of both upper motor neurons (UMNs) in the motor cortex and lower motor neurons (LMNs) in the brainstem and spinal cord. As these neurons deteriorate, individuals may suffer from atrophy, muscle weakness, and ultimately paralysis, while their cognitive abilities typically remain unaffected [113].

Its etiology remains unknown, which makes diagnosis complex and, in some cases, time-consuming due to the lack of specific biomarkers, which can lead to delays in identifying the disease, especially in its early stages [114]. Numerous deleterious processes have been suggested to play a role in the molecular mechanisms of the disease, and these include different types of cellular abnormalities, such as intranuclear and cytosolic protein deposits and RNA aggregates, disorders of protein degradation mechanisms, such as genetic alteration of the protein called mitochondrial antioxidant enzyme superoxide dismutase 1 (SOD1), in which it is possible to describe the mechanism of oxidative stress and its relationship with neuronal death, excitotoxicity mediated by excess activation of the glutamate receptor, mitochondrial dysfunction, and microglial activation [115].

However, despite extensive research, a cure for ALS has not yet been found, and current treatments are aimed at managing symptoms and slowing disease progression. Understanding the underlying molecular mechanisms of ALS remains a critical step in developing targeted therapies that could provide a curative treatment for this disease [116].

#### Targeting the GBA in ALS: Emerging Therapeutic Strategies

Increasing studies suggest that ALS patients exhibit gut dysbiosis, characterized by reduced beneficial bacteria and increased pathogenic genera, which correlates with systemic inflammation and neuroinflammation. From this point of view, treatment strategies and interventions that target the GBA and gut microbiota could offer potential benefits (Table 4). In ALS models, Song et al. illustrated that GOS-rich yogurt improved mitochondrial function, reduced muscle atrophy, and slowed disease progression. It also lowered neuroinflammation and motor neuron degeneration [117]. Notably, these effects were associated with modulation of gut microbiota and suppression of proinflammatory cytokines, suggesting a GBA-mediated mechanism. A study investigating the effect of *Lactaseibacillus rhamnosus* HA-114 on neurodegeneration found a delay in neurodegeneration in FUS and TDP-43 worm models of ALS via the restoration of energy metabolism and mitochondrial function, indicating a potential gut-mitochondrial axis involved in neuronal preservation [118]. While these findings are promising, they remain confined to preclinical models, and translational studies in mammalian systems or humans are still required. In contrast, clinical investigation on AJ3 probiotic bacteria in ALS patients has provided early human-based evidence for immunomodulatory effects. In a pilot study, administration of AJ3 significantly increased interleukin-10 (IL-10) and reduced interferon-gamma (IFN-γ) levels, as ALS patients show high levels of IFN-γ from immune cells, contributing to immune-related issues. AJ3 probiotic bacteria help to alleviate autoimmunity in ALS by regulating cytotoxic immune responses and protecting motor neurons [119]. In TDP-43 transgenic mice, treatment with butyrate or probiotics improved the condition of intestinal motility, reducing permeability, and decreasing inflammatory markers [120]. These effects suggest that postbiotics can restore intestinal homeostasis and attenuate neuroinflammatory cascade linked to ALS pathogenesis. Further supporting this, Zhang et al. found that 2% butyrate in drinking water improved gut microbial balance, strengthened the gut barrier, delayed disease progression, and extended lifespan in SOD1-G93A mice, while reducing Paneth cell accumulation and mutant protein aggregation [121]. These findings suggest that postbiotics have therapeutic potential mainly due to their ability to address gut-related issues and inflammation. Research carried out by Ogbu et al. illustrated that sodium butyrate modified carbohydrate and amino acid metabolism, decreased microglia in the spinal cord, and lowered proinflammatory markers IL-7 and LPS [122]. Ryu et al. linked the effect of sodium phenylbutyrate’s neuroprotective effects to the upregulation of anti-apoptotic genes like NF-kB and Bcl-2, inhibiting the activation of caspase and, frequently, motor neuron death. While phenylbutyrate treatment alone slowed ALS progression [123], the combination of sodium butyrate with riluzole improved survival, body weight, and grip strength in ALS mice [124]. Future directions for ALS treatment focus on modulating the gut microbiome using probiotics or prebiotics to improve immune balance and reduce disease-associated neuroinflammation. Promising research is also underway on the use of microbiome transplantation (FMT) as a method for transferring a healthy microbiome to a patient to improve the interaction between the gut and the nervous system. Additionally, the role of SCFAs, particularly butyrate, in supporting neuronal function and reducing inflammation is being investigated. The hope for the future is to use these approaches in conjunction with conventional drug therapies, and perhaps even personalized to each patient based on microbiome and genetic analyses. Although these ideas are still in the early stages of research, the gut–brain axis may represent a new avenue for understanding and modifying the course of the disease in the future.

### 4.5. Overview of HD

HD is a progressive neurodegenerative disease with an autosomal dominant inheritance pattern that occurs due to a mutation in the gene that encodes the Huntingtin (Htt) protein. It is characterized by progressive motor dysfunction, cognitive decline, and psychiatric disturbances [125]. The mutation in the *HTT* gene located on chromosome 4 results in an expansion of the cytosine–adenine–guanine CAG trinucleotide sequence, responsible for the transcription of glutamine residues at the amino terminus of the HTT protein [126]. It is worth noting that HTT is normally expressed in neuronal and non-neuronal cells and plays a fundamental role during embryonic development, and inactivation of the gene that encodes the Htt protein results in embryonic death [127]. HD is characterized by motor disorders associated with the loss of voluntary movement coordination [127]. Although mutated Huntingtin (mHTT) is expressed throughout life, in most cases the first symptoms appear only in adulthood, between 35 and 50 years of age [128], and are fatal 15 to 20 years after the appearance of the first motor symptoms [129].

The neuropathology of HD is characterized by atrophy of the striatum [127], which is related to the planning and execution of motor behavior [130]. In the more advanced stages of the disease, intracellular dysfunction induced by mHTT leads to the degeneration of important neuronal pathways and nearly complete loss of GABAergic medium spiny neurons, cerebral cortex, and other brain regions [131]. The mHTT also disrupts another cellular process, including autophagy and mitochondrial function, contributing to neurodegeneration [132].

#### Modulating the GBA in HD: Insights into Therapeutic Approaches

In HD also, the dysregulation of GBA axis may exacerbate neurodegeneration [133].

In a study involving 42 HD gene expansion carriers (19 diagnosed and 23 premanifest) with 36 age- and gender-matched healthy controls, cognitive tests, and 16S rRNA sequencing of fecal samples were used (Table 5). It was discovered that the intestinal microbial composition of experimental patients with HD is significantly different from that of healthy controls. This study provides preliminary evidence that HD is associated with intestinal flora disorders, indicating that intestinal microbes play an important role in HD [134]. Kong et al. compared the intestinal microbes of healthy mice and genetically modified experimental mice with HD and found that the intestinal microbial composition of sick mice was significantly different from that of healthy mice. When sick mice reached 12 weeks of age, the number of Bacteroidetes in their intestines increased, while the number of Firmicutes decreased proportionally. In addition, the diversity of intestinal microbes in male sick mice increased, but their intestinal function gradually failed. They ate a lot but did not gain much weight, and the early symptoms of HD also appeared at this time [135].

Researchers successfully tested a dietary treatment with a fatty acid, triheptanoin, in 10 patients with an early form of the disease. After one month, they observed a reduction in abnormalities in the patients’ brain metabolism [136]. A key player in brain metabolism, cholesterol, is also a path that has been studied in HD. Cholesterol metabolism is impaired in HD, with different studies indicating reduced cholesterol levels in the brains of HD patients and models [137].

In addition, in a randomized controlled trial on 41 HD gene expansion carriers, the study found that the HD group exhibited altered gut microbiome diversity, indicating gut dysbiosis compared to healthy controls but, when assessing a 6-week probiotic intervention, results showed no improvement in gut dysbiosis, cognition, mood, or gastrointestinal symptoms, highlighting the need for further exploration of gut-targeted therapies [138].

FMT involves the transfer of fecal material from a healthy donor to an affected individual to restore microbial balance. Preliminary animal studies have shown that FMT can ameliorate cognitive deficits and motor dysfunction by re-establishing a more favorable microbial ecosystem, particularly in females [139]. While preclinical results are promising, there is a lack of clinical trials specifically examining FMT in HD patients.

Pharmacological modulation of the GBA is another promising target. Drugs that modulate gut-derived metabolites or inflammatory pathways may help alleviate the symptoms of HD. The imbalance in kynurenine (KP) leads to elevated levels of neurotoxic metabolites, contributing to neurodegeneration and inflammation [140]. Inhibitors of KP enzymes, such as kynurenine monooxygenase (KMO), have shown promise in reducing neuroinflammation and oxidative stress. Ablation of KMO in R6/2 mice normalized the impaired kynurenine pathway genes and increased levels of the neuroprotective kynurenic acid while reducing levels of the neurotoxic 3-hydroxykynurenine in the brain and periphery. Elevated pro-inflammatory plasma cytokines (TNF-α, IL-1β, IL-4, and IL-6) were also normalized after KMO deletion, indicating reduced peripheral inflammation. However, these metabolic and inflammatory improvements did not translate into improvements in the behavioral phenotype associated with HD. Absence of KMO did not negatively affect the R6/2 phenotype, suggesting that no compensatory mechanisms were activated. Overall, targeting KMO may offer therapeutic potential for modulating inflammation and metabolism in Huntington’s disease [141].

Future directions in the treatment of HD point to the importance of targeting the GBA as a promising therapeutic avenue. These directions include modulating the gut microbiome with probiotics and prebiotics to reduce neuroinflammation and improve cognitive and motor functions. Diet, particularly the Mediterranean diet, is also important in promoting microbial diversity and increasing the production of short-chain fatty acids that support brain health. Fecal microbiome transplantation is also being explored to restore microbial balance and improve symptoms, particularly in animal models. Furthermore, modulating inflammatory and metabolic pathways, such as the abovementioned kynurenine pathway, is considered as a means of reducing neurotoxicity and decreasing inflammatory cytokines. The effects of fatty acids such as triheptanoins on improving brain metabolism are being investigated. Together, these approaches open new avenues for treating HD by targeting the complex interaction between the gut and the brain.

**Table 5:** Studies evaluating GBA in HD.

Intervention	Key Findings—Related Targets and Pathways	Model/Limitations	Effect on HD	Ref.
Analysis of gut microbiota composition in 42 HD gene expansion carriers (19 manifest and 23 premanifest) compared to 36 age- and gender-matched healthy controls	HD gene expansion carriers exhibited significantly reduced alpha diversity (species richness and evenness; *p* = 0.001) and distinct beta diversity (microbial community structure; *p* = 0.001) compared to controls. Taxonomic analyses revealed shifts in microbial phyla and families, alongside differences in functional pathways and enzyme profiles. Specific gut microbes were linked to cognitive performance and clinical outcomes, suggesting microbiota involvement in HD pathophysiology	Human cohort/Small sample size, Lack of longitudinal data, No functional or mechanistic insights	NOT AVAILABLE	[134]
Utilization of 16S rRNA amplicon sequencing to characterize the gut microbiome in R6/1 transgenic HD mice compared to wild-type littermate controls at 12 weeks of age	HD mouse models showed gut dysbiosis characterized by increased Bacteroidetes, reduced Firmicutes, sex-specific microbial diversity changes, and physiological alterations, including impaired weight gain, motor deficits, and increased fecal water content	Transgenic mice model/Mouse model limitations, small Sample size, Limited scope of investigation, No mechanistic or metabolic follow-up	The observed gut dysbiosis in HD mice was associated with systemic physiological changes, including weight loss and motor deficits, indicating that alterations in the gut microbiome may contribute to the progression and severity of Huntington’s disease symptoms	[135]
Administration of triheptanoin (1 g/kg/day) for 1 month to early-stage HD patients	Triheptanoin improved brain energy metabolism in HD patients by restoring Pi/PCr ratios during stimulation and supporting mitochondrial function through its anaplerotic action on the Krebs cycle	Human patients/Small patient sample size, Lack of placebo control group, Open-Label Design, Metabolic improvements not directly linked to clinical outcomes	Improved brain energy metabolism was associated with stabilization or improvement in motor function, suggesting potential therapeutic benefits in slowing HD progression	[136]
A 6-week double-blind, placebo-controlled trial tested probiotics on 41 HD gene expansion carriers (HDGECs), consisting of 19 early manifest and 22 premanifest individuals, compared to 36 healthy controls	HDGECs exhibited altered gut microbiome diversity compared to healthy controls, indicating gut dysbiosis; however, probiotic treatment failed to improve microbial composition or clinical outcomes such as cognition, mood, and gastrointestinal symptoms	Human randomized controlled trial/No metabolomic or mechanistic data	The probiotic intervention did not produce measurable benefits in gut microbiota composition or clinical symptoms in HDGECs. However, the study underscores the potential of the gut as a therapeutic target in HD, warranting further exploration of alternative gut-targeted interventions	[138]
Fecal microbiota transplantation (FMT) from wild-type mice into HD mice	FMT from wild-type mice into HD mice restored gut microbiota diversity and composition. It improved cognitive function, particularly in female HD mice, while male HD mice showed less efficient FMT engraftment, potentially due to greater gut microbial instability	Transgenic mice model/Small sample size limits generalizability, Mechanistic pathways linking microbiota changes to cognition unclear	The findings suggest that FMT can modulate gut microbiota and improve cognitive function in HD mice, highlighting the potential of gut–brain axis interventions in neurodegenerative diseases	[139]
Genetic ablation of kynurenine 3-monooxygenase (KMO) in R6/2 mice	In R6/2 mice, significant increases in plasma cytokines (TNFα, IL1β, IL4, IL6, and IL10) were observed compared to wild-type controls, but KMO ablation normalized these elevated levels to near wild-type levels, while also increasing neuroprotective kynurenic acid (KYNA) and decreasing neurotoxic 3-hydroxykynurenine (3-HK) in the brain and periphery	Transgenic mice model/animal model limitations, focus on single enzyme limits broader pathway understanding	While KMO ablation led to normalization of inflammatory cytokine levels and favorable shifts in kynurenine pathway metabolites, it did not result in improvements in behavioral phenotypes or disease progression in R6/2 mice	[141]

## 5. Neurotrauma

### 5.1. Pathophysiological Processes of TBI

TBI is defined as an alteration in brain function caused by an external force. These alterations may include loss or decrease in the level of consciousness, memory loss and changes in mental status in the acute phase, as well as neurological deficits such as paresis or plegia, aphasia, dyspraxia, sensory and body balance alterations [142].

Neuroinflammation is the major pathological process during secondary TBI injury [143]. The inflammatory response involves many cell types belonging to the CNS [144]. This response includes local signaling from neurons, glia, and recruited peripheral immune cells, which induce the inflammatory cascade [145]. The glial cell response includes secretion of pro- and anti-inflammatory cytokines, chemokines, and growth factors; formation of a barrier around the injured areas; phagocytosis of dead cells and cell debris; and modulation of cellular responses [146].

It is now known that secondary damage from TBI is not limited to the CNS, since it was already established that it can cause damage to the heart, liver, spleen, and gastrointestinal tract [147,148,149,150]. Among the intestinal dysfunctions observed in TBI victims, one of the most notable is the alteration of the microbiota, which has been reported even 20 years after the primary injury [151]. In experimental models, TBI has also been associated with mucosal damage and breakdown of the intestinal barrier [150]. Such events can lead to endotoxemia and systemic inflammation, which may involve other organs in the response to secondary damage, such as the spleen [152].

#### GBA in TBI: Insights into Novel Therapies

The GBA plays a significant role in how TBI develops and progresses; indeed, it was demonstrated that antibiotic-induced gut microbial dysbiosis after TBI showed decreased neuroinflammation, as evidenced by reduced microglial activation and cortical damage, with alteration in the intestinal morphology and no effect on SCFA production [153]. However, gut microbial dysbiosis after TBI leads to increased neuroinflammation and reduced neurogenesis, impairing cognitive functions and recovery (Table 6). Additionally, TBI-induced dysbiosis also alters the immune response by reducing the infiltration of immune cells, which is crucial for effective brain repair, and by affecting microglial dynamics, which further compromises neuronal survival and overall brain health [154]. To manage this challenge, several therapeutic strategies have been demonstrated. Supplementation with specific strains of beneficial bacteria (probiotics) or indigestible fibers (prebiotics) that promote their growth has shown the potential to improve cognitive outcomes and potentially influence metabolic regulation and reduce neuroinflammation, even though their effect on pro-inflammatory markers was limited, which is elevated in TBI conditions [155].

Inulin supplementation after three months post-injury effectively improves brain impairment by modulating the gut microbiome to increase beneficial bacteria, producing anti-inflammatory SCFAs, and restoring cerebral blood flow in affected brain regions, which collectively support recovery from mild TBI [156].

Diets rich in omega-3 fatty acids, polyphenols, and fiber have been associated with improved gut health and reduced neuroinflammation after TBI. Omega-3s have been shown to suppress pro-inflammatory microglial transformation and activate neuroprotective pathways, reduce cerebral edema and inflammatory cytokine levels, such as NF-κB, IL-1β, IL-6, and TNF-α by targeting the PPARγ/NF-κB signaling pathway, indicating a decrease in neuroinflammation, thereby improving neurological outcomes [157]. Similarly, ketogenic diets, which are high in fat and low in sugar, have garnered attention for their neuroprotective effects, potentially mediated through reducing neuroinflammation, modulating gut microbiota, and ketone body production [158].

Recent studies have highlighted the potential role of fecal FMT in addressing neurological impairments and inflammatory problems following TBI. FMT has been shown to alleviate neurological deficits associated with TBI by modulating the gut–brain axis and reducing neuroinflammation [159].

The following studies have focused on how transplantation of donor microbiota alleviates neuroinflammation after TBI. They found that transplantation of donor microbiota not only selectively improved neurological function but also neuroinflammatory responses in TBI mice. This was achieved by resuming the maintenance of the gut microbiota, which in turn inhibited microglial activation and reduced the production of inflammatory cytokines such as TNF-α [159].

In addition, transplantation of donor microbiota increased synaptic plasticity by increasing proteins such as PSD-95 and synapsin I, promoting the proliferation of regulatory T cells (Tregs) in the spleen, further contributing to an anti-inflammatory environment [160]. Other studies showed that transplantation of microbiota from healthy donors promoted good recovery in TBI rats by altering the distribution of gut microbiota composition and increasing the integrity of the gut and brain barrier. These findings underscore the therapeutic potential of FMT in addressing TBI-induced neuroinflammation through the gut–brain axis [161,162]. Future research should explore the role of the intestinal flora in brain–gut communication to promote the development of new TBI treatment interventions and understand the roles of SCFAs, tryptophan metabolism, and serotonin pathways in neuroprotection. Moreover, studies on human populations are needed to guide the development of interventions for TBI-induced metabolic diseases with the intestinal flora as a target. The causal relationship between gut flora and TBI and the exact mechanism of gut flora metabolites in the development of TBI need to be further investigated to provide preclinical evidence on the use of gut flora in the treatment of TBI, Additionally, personalized approaches based on individual microbiome profiles may enhance recovery and guide precision treatment strategies for TBI.

**Table 6:** Studies assessing GBA in TBI.

Intervention	Key Findings—Related Targets and Pathways	Model/Limitations	Effect on TBI	Ref.
Administration of broad-spectrum antibiotics (vancomycin, neomycin-sulfate, ampicillin, and metronidazole) to induce gut microbial dysbiosis in mice before, during, and after TBI	Antibiotic-induced dysbiosis modulated the immune response after TBI by reducing Ly6C^high^ monocyte and T cell infiltration into the brain, increasing microglial activation (TLR4, MHCII), impairing neurogenesis in the dentate gyrus, and leading to cognitive deficits evidenced by altered fear memory behavior	Mouse Model/Lack of control groups, Variability in results, Limited behavioral assessment	Induced dysbiosis post-TBI led to increased neuronal loss in the hippocampal CA3 region, persistent microglial activation driving chronic neuroinflammation, and long-term cognitive impairments, especially in fear memory performance	[154]
Male C57BL/6J mice were administered a broad-spectrum antibiotic cocktail (ampicillin, gentamicin, metronidazole, and vancomycin) via oral gavage for 2 days to deplete gut microbiota, followed by traumatic brain injury (TBI) induction using CCI model	Antibiotic treatment led to significant shifts in gut microbiota diversity and abundance during both acute and chronic phases post-TBI, and to reduced neuroinflammation, marked by decreased microglial activation, resulting in improved neurological outcomes	Mouse model/Short duration of observation, Mouse model limits direct human translation, Functional impacts unclear	Antibiotic-induced microbiota depletion improved neurological recovery post-TBI, indicating a neuroprotective effect likely mediated through modulation of the brain–gut axis and attenuation of neuroinflammatory responses	[153]
Administration of probiotics: *Lactobacillus helveticus* and *Bifidobacterium longum*	Probiotic treatment reversed TBI-induced reductions in hippocampal mRNA expression of CaMKII and CREB, improved glucose metabolism, altered gut microbiota composition, and partially restored disrupted hepatic lipid profiles, while having no significant effect on BDNF mRNA levels or neuroinflammatory markers	Mouse model/Pre-treatment design, not including both sexes in the experimental design, No motor/neurological functional analysis, Translational gap	Probiotic treatment in TBI mice improved memory performance, glucose tolerance, and neuroplasticity markers (CaMKII and CREB), but did not significantly reduce TBI-induced pro-inflammatory cytokine levels	[155]
Administration of an 8% inulin-enriched diet to male mice, starting 3 months post-injury (mpi) and continuing for 8 weeks	Inulin supplementation enhanced gut microbiota by increasing beneficial bacteria and SCFAs, while also restoring cerebral blood flow in both hippocampi and left thalamus and improving white matter integrity, as indicated by MRI assessments	Mouse model/Mouse model limits direct human translation, Lack of motor and cognitive testing, small sample size, Clinical relevance requires human trials	The findings suggest that inulin supplementation during the chronic phase post-mild TBI can modulate the gut microbiota, leading to improved brain vascular and structural integrity, as well as enhanced cognitive function	[156]
Administration of Omega-3 Polyunsaturated Fatty Acids (ω-3 PUFAs), specifically docosahexaenoic acid (DHA) and eicosapentaenoic acid (EPA), in a mouse model of TBI	ω-3 PUFAs reduced levels of pro-inflammatory cytokines (TNF-α, IL-1β, IL-6) and increased anti-inflammatory cytokine IL-10, shifted microglial activation from pro-inflammatory M1 phenotype to anti-inflammatory M2 phenotype, and inhibited the HMGB1/NF-κB signaling pathway through SIRT1-mediated deacetylation, reducing neuroinflammation, and suppressed necroptosis by downregulating RIP1 and RIP3 expression	Mouse model/lack of behavior tests, Translational gap to humans	The findings suggest that omega-3 PUFA supplementation can mitigate early brain injury following TBI by modulating inflammatory responses and inhibiting necroptotic pathways, thereby improving neurological outcomes	[157]
Transplantation of gut microbiota from healthy donor rats into male rats with TBI induced by gas explosion	FMT restored microbial diversity and balance, correcting dysbiosis caused by TBI, enhanced expression of tight junction proteins (Claudin-1, Occludin, ZO-1) to improve intestinal barrier integrity, improved BBB function by reducing permeability and potential neuroinflammation, and increased levels of regulatory T cell (Treg)-related factors (IL-10, PD-1, FoxP3), indicating an anti-inflammatory response.	Rat model/Lack of functional analysis, Focus on male rats, Rat model may not fully replicate human TBI.	The findings suggest that FMT can mitigate cognitive impairments following TBI by restoring gut microbiota balance, enhancing barrier functions, and modulating immune responses, highlighting its potential as a therapeutic strategy for TBI-related cognitive deficits.	[159]
Administration of fecal microbiota from healthy donor mice to TBI-induced mice.	FMT inhibited microglial activation and reduced pro-inflammatory cytokine TNF-α levels, increased expression of synaptic proteins PSD-95 and synapsin I, indicating improved synaptic integrity, and enhanced population of regulatory T cells (Tregs) in the spleen, suggesting modulation of systemic immune response	Mouse model/Mouse model limits human applicability, short intervention duration, the number of mice used doesn’t specified, No sex-specific reporting	The findings suggest that FMT can modulate the gut–brain axis to suppress neuroinflammation and promote neurological recovery following TBI	[160]
Administration of fecal microbiota from healthy donor rats to male Sprague-Dawley rats subjected to CCI to induce TBI. FMT was performed for 7 consecutive days	TBI induced significant changes in gut microbiota diversity and composition; FMT restored these alterations, TBI increased levels of trimethylamine (TMA) in feces and trimethylamine N-oxide (TMAO) in the brain and serum; FMT reduced these levels, and TBI decreased antioxidant enzyme methionine sulfoxide reductase A (MsrA) and increased oxidative stress markers; FMT reversed these effects	Rat model/Limited sample size, Lack of control over microbiota composition, No motor behavior data, Male-only subjects	The findings suggest that FMT can restore gut microbiota balance and alleviate neurological deficits following TBI, potentially through modulation of the gut–brain axis and reduction of oxidative stress	[161,162]
Administration of fecal microbiota from healthy donor mice to C57Bl/6 mice subjected to severe TBI via controlled cortical impact. FMT was performed weekly starting 1 h post-injury	TBI led to significant reductions in species richness and evenness; FMT restored these parameters, indicating reversal of dysbiosis. Histological analysis showed that FMT reduced microglial activation, as evidenced by decreased Iba1 expression, suggesting attenuation of neuroinflammatory responses. MRI assessments demonstrated that FMT mitigated ventriculomegaly and preserved white matter integrity, as indicated by improved fractional anisotropy values	Mouse model/Small sample size, Short-term observations, Animal model limits direct human applicability	The findings suggest that FMT can effectively restore gut microbiota balance and alleviate both functional and structural deficits following TBI, potentially through modulation of the gut–brain axis and reduction of neuroinflammation	[161,162]

### 5.2. Neurobiology of SCI

SCI is a devastating neurological condition characterized by a complex cascade of molecular, cellular, and systemic events that extend far beyond the initial mechanical insult [163]. One of the earliest and most prominent events following SCI is the disruption of the blood–spinal cord barrier (BSCB), which leads to a rapid infiltration of peripheral immune cells and the release of pro-inflammatory mediators such as TNF-α, IL-1β, and IL-6 [164]. This inflammatory milieu, coupled with the activation of resident microglia and astrocytes, exacerbates neuronal and oligodendrocyte death and impairs endogenous repair mechanisms [164]. At the same time, glutamate-mediated excitotoxicity, driven by excessive activation of NMDA and AMPA receptors, contributes to calcium overload and mitochondrial dysfunction, amplifying ROS production and lipid peroxidation [163,165]. Another critical aspect of SCI neurobiology is the dysregulation of systemic homeostasis, particularly involving the autonomic nervous system [166]. The loss of supraspinal control can disrupt cardiovascular, gastrointestinal, and immune function, setting the stage for chronic systemic inflammation and susceptibility to infections [166]. Importantly, recent studies have highlighted the role of the GBA in shaping the neuroimmune response post-SCI. Injury-induced autonomic dysfunction alters gastrointestinal motility, secretory activity, and mucosal immunity, contributing to dysbiosis and increased intestinal permeability [167]. This, in turn, facilitates the translocation of microbial products such as LPS into the circulation, which can act as systemic immune triggers and further exacerbate neuroinflammation at the injury site [168]. Moreover, alterations in microbial metabolites, including a reduction in neuroprotective short-chain fatty acids, may impair anti-inflammatory signaling and hinder recovery [168]. Thus, the neurobiology of SCI is not confined to the lesion epicenter but involves a systemic response. in which peripheral and central mechanisms interact in a dynamic and bidirectional manner. This highlights the critical involvement of the GBA in modulating both injury progression and recovery. Given the pivotal role of gut-derived inflammatory signals in the post-SCI neuroinflammatory response, therapeutic strategies targeting the GBA offer an exciting avenue for mitigating secondary injury and promoting neurorepair. These approaches may include restoring gut microbiota balance, reducing intestinal permeability, and modulating immune responses, thereby offering the potential for novel, multifaceted treatments to enhance recovery and quality of life following SCI.

#### Therapeutic Strategies for GBA Modulation in SCI

Several innovative strategies are being explored to regulate the complex interplay between the gut, immune system, and nervous system in SCI (Table 7).

One of the most promising therapeutic approaches involves the use of Pectin-Zein-Indole-3-Proprionate (IPA) nanoparticles, an innovative formulation that has shown significant benefits in experimental SCI models [169]. IPA, a metabolite produced by gut bacteria, has been demonstrated to promote axonal regeneration; however, its short half-life limits its therapeutic potential. By utilizing Pectin-Zein-IPA nanoparticles, researchers have enhanced its bioavailability, thereby prolonging its beneficial effects [169]. Studies in SCI animal models have yielded promising results, where Pectin-Zein-IPA treatment not only improved motor function recovery but also inhibited oxidative stress, stimulated axonal regeneration, and activated protective signaling pathways such as AKT/Nrf-2 [169]. Furthermore, the treatment modulated the gut microbiome by reducing the accumulation of L-methionine, a metabolite involved in neuroinflammation [169]. The Pectin-Zein-IPA nanoparticles also altered the composition of the gut microbiome, decreasing the abundance of pro-inflammatory bacteria, such as *Clostridia-UCG-014* and *Shewanella,* while increasing the prevalence of beneficial bacteria like *Parasutterella* [169]. This dual effect on both the microbiome and the injured spinal cord underscores the potential of Pectin-Zein-IPA nanoparticles as a therapeutic strategy for SCI [169].

Another innovative therapeutic strategy focuses on the modulation of hydrogen sulfide (H_2_S) production in the gut. H_2_S is a toxic gas that can accumulate in the intestine following traumatic stress on the nervous system, and recent studies have highlighted its role in neuropathic pain, a common consequence of SCI. Excessive production of H_2_S in the gut has been associated with the activation of NMDA receptors in the spinal cord, contributing to pain hypersensitivity and neuroinflammation [170]. In chronic nerve injury models, inhibiting H_2_S production significantly alleviated pain and reduced neuroinflammation, suggesting that the GBA may play a pivotal role in the development of neuropathic pain [170]. Thus, intervening in H_2_S production in the gut could represent a novel therapeutic avenue for treating pain and inflammation following SCI, offering a more targeted approach to this debilitating condition.

In addition, the use of exoskeleton-assisted walking (EAW) has emerged as an innovative rehabilitation technique for SCI patients, with significant potential in modulating the GBA. While the primary goal of EAW is to improve mobility and functional recovery, latest studies have shown that the use of exoskeletons can also enhance intestinal function. In a pilot study conducted with patients with motor-complete paraplegia, subjects in the EAW group showed significant improvements in bowel management, such as increased bowel evacuation frequency and reduced reliance on external assistance. like manual digital stimulation or medications [171]. The underlying mechanism for these improvements appears to be related to changes in the gut microbiome. Specifically, EAW led to an increase in beneficial bacteria, such as *Faecalibacterium* and *Bifidobacterium*, known for their role in maintaining gut health and modulating inflammation [171]. The study suggests that EAW may help recover neurogenic bowel dysfunction (NBD), a common complication in SCI, by regulating the gut microbiome and enhancing the communication between the gut and the brain [171].

Another emerging therapeutic target in SCI is the PPARα, particularly in the context of neuropathic pain. PPARα is a nuclear receptor involved in lipid metabolism and inflammatory regulation, and its expression has been linked to gut microbiome modulation.

In a study examining the effects of PPARα on neuropathic pain in chronic nerve injury models, it was found that PPARα activation altered the gut microbiome composition, reduced pain sensitivity, and promoted anti-inflammatory responses [172]. PPARα agonists increased the prevalence of beneficial gut bacteria and directed spinal microglia polarization towards the protective M2 phenotype, suggesting that PPARα may represent a promising therapeutic target for modulation of both the gut microbiome and spinal cord inflammation in SCI, thereby alleviating pain and promoting recovery [172].

Furthermore, GV-971, a drug originally developed for treating AD, has recently been explored for its potential therapeutic effects in SCI [173]. GV-971 works by modulating the GBA and reducing neuroinflammation, both of which are crucial in the pathophysiology of SCI [173]. In murine models of neuromyelitis optica, GV-971 has been shown to reduce neuroinflammation, protect against spinal cord injury, and restore the balance of the gut microbiome [173]. Additionally, GV-971 ameliorated peripheral inflammation and metabolic disorders, offering a comprehensive approach to SCI management [173]. These findings suggest that GV-971 could be an effective therapeutic agent for SCI patients, simultaneously addressing both neuroinflammation and gut microbiome imbalances. Overall, these emerging therapeutic strategies highlight the significant role of the GBA in SCI recovery. By targeting gut microbiome composition, modulating inflammation, and enhancing neuroprotective signaling, researchers are uncovering novel ways to promote spinal cord repair, alleviate pain, and improve overall function in SCI patients. These multifaceted approaches, which combine microbiome-based therapies, exoskeleton-assisted rehabilitation, and pharmacological interventions, have the potential to revolutionize SCI treatment. Future research concentrates on the activity of the gut–brain axis as a potential target of new treatments in SCI, because a gut microbiome is involved in the regulation of the inflammatory response, including its influence on neuronal recovery following injury. One promising approach is the development of therapies that aim to modulate imbalanced gut microbiota, such as the application of probiotics, prebiotics, and fecal microbiota transplantation. These interventions are aimed at increasing SCFAs, which help in attenuating inflammation. The effect of certain antibiotics, like minocycline, is also under investigation, as it has anti-inflammatory properties, enhances microbial diversity, and has limited efficacy on motor recovery. Moreover, there is growing interest in natural therapies such as melatonin and plant phenols, which may reduce the activation of inflammation associated with the gut microbiome. Furthermore, AI can be employed to facilitate the discovery of microbiome-related therapies and enhance the comprehension of drug-microbiome interactions, leading to promising avenues for treating the psychological and neurological complications associated with SCI.

**Table 7:** Articles evaluating GBA in SCI.

Intervention	Key Findings—Related Targets and Pathways	Model/Limitations	Effect on SCI	Ref.
Oral administration of nanoparticles composed of pectin and zein encapsulating indole-3-propionic acid (IPA) to mice with spinal cord injury	The intervention reduced pro-inflammatory cytokines (IL-6, TNF-α, IL-1β) and shifted microglial polarization from the M1 to M2 phenotype, indicating attenuation of neuroinflammation. It also activated the AKT/Nrf-2 signaling pathway, thereby enhancing the expression of antioxidant proteins such as HO-1, NQO1, and SOD2, which contributed to a reduction in oxidative stress. Additionally, it modulated gut microbiota composition by decreasing L-methionine-producing bacteria (e.g., *Clostridia_UCG-014*) and increasing beneficial bacteria (e.g., *Parasutterella*), leading to reduced L-methionine accumulation. Furthermore, intestinal barrier function was improved through the upregulation of tight junction proteins (ZO-1, Occludin) and inhibition of the NF-κB signaling pathway	Mouse model/Small sample size, Nanoparticle safety and long-term effects need assessment, short half-Life of IPA, Sex not reported	The findings suggest that Pectin-Zein-IPA nanoparticles can effectively promote functional recovery and alleviate neuroinflammation after spinal cord injury by modulating gut microbiota, reducing oxidative stress, and enhancing neuronal regeneration	[169]
Increased generation of H_2_S in the colon following chronic constriction injury (CCI) in rodents	Elevated colonic H_2_S levels lead to activation of spinal N-methyl-D-aspartate (NMDA) receptors, contributing to neuropathic pain. This pain response is attenuated by the administration of H_2_S synthesis inhibitors or NMDA receptor antagonists (e.g., MK-801), highlighting the pivotal role of H_2_S-NMDA receptor interaction in pain modulation	Mouse model/Lack of direct mechanistic link, Sex unspecified, Translational applicability unclear	Colon-derived H_2_S induces neuropathic pain behaviors in rodents, suggesting that targeting its production or spinal NMDA receptor activation could offer potential therapeutic strategies for pain management	[170]
Implementation of EAW training in patients with motor-complete SCI (T2–L1) to assess its impact on bowel function and gut microbiota	EAW training altered the abundance of intestinal flora, notably increasing beneficial bacteria, suggesting gut microbiota modulation, which may be linked to improvements in intestinal function through changes in the brain–gut axis, though the exact mechanisms remain unclear	Human Pilot clinical study/Small sample size, Pilot study design; no control group, Short duration	The findings suggest that EAW may serve as a promising intervention to alleviate bowel dysfunction in individuals with motor-complete spinal cord injuries, potentially through modulation of the gut microbiota and the brain–gut axis	[171]
Administration of a PPARα agonist (GW7647) and antagonist (GW6471) in mice with chronic constriction injury (CCI) to assess the role of PPARα in neuropathic pain	PPARα activation altered the abundance, homogeneity, and composition of the gut microbiome, as revealed by 16S rRNA gene sequencing, and led to significant changes in spinal cord metabolites, including nicotinamide, benzimidazole, eicosanoids, and pyridine, that correlated with specific gut microbiota alterations; additionally, it promoted M2-type microglia polarization, thereby reducing neuroinflammation in the spinal cord	Mouse model/Small sample size, Limited scope of microbial analysis, Sex unspecified	The findings suggest that PPARα plays a crucial role in modulating neuropathic pain by influencing gut microbiota composition, spinal cord metabolite profiles, and microglial activation states. Targeting PPARα may offer a promising therapeutic strategy for managing neuropathic pain	[172]
Oral administration of GV-971, a marine-derived oligosaccharide approved in China for mild-to-moderate Alzheimer’s disease, to NMOSD murine models induced by NMO-IgG and complement	GV-971 remodeled gut microbiota composition and restored microbial balance disrupted by NMOSD. It reduced peripheral inflammation and metabolic disorders, as shown by cytokine and metabolomics analyses. Histological evaluations revealed reduced neuroinflammation and neural injury in the spinal cord. These findings suggest GV-971 exerts therapeutic effects through gut-immune-CNS axis modulation	Mouse model/Mixed disease induction strategies which limit clarity on mechanism, Focus on young and aged mice	The findings suggest that GV-971 may attenuate the progression of NMOSD by modulating the gut microbiota and reducing peripheral and central inflammation	[173]

## 6. Conclusions and Future Perspectives

In this review, we have evidenced the intricate relationship between the gut and the brain as a critical determinant in the pathophysiology of neurodegenerative and neurotraumatic disorders. In particular, this review highlights the multifaceted nature of the GBA, encompassing immune, endocrine, and neuronal pathways, and its deep entanglement with CNS health, also underscoring the role of gut dysbiosis and its pro-inflammatory milieu in exacerbating CNS disease onset, progression, and severity. Moreover, emerging interdisciplinary fields such as metabolomics and neuroimmunology are increasingly shedding light on the complex molecular and cellular mechanisms underlying these interactions. Metabolomic profiling allows for the identification of microbiota-derived metabolites that modulate neuroinflammation and neurodegeneration, while neuro-immunological studies provide critical insights into the bidirectional communication between peripheral immune responses and CNS homeostasis. Thus, targeting the gut microbiome to modulate neuroinflammation and support neuro-regeneration represents a paradigm shift in the treatment of disorders. such as AD, PD, MS, ALS, HD, and CNS traumas, like TBI and SCI. Probiotic supplementation, dietary interventions, and microbiota-derived metabolite therapies, particularly those involving SCFAs, stand out as promising, non-invasive strategies capable of restoring microbial balance and exerting neuroprotective effects. However, despite encouraging preclinical and early clinical data, substantial gaps remain. The heterogeneity of microbiome compositions across individuals, disease stages, and geographic populations poses a challenge to standardized therapeutic approaches. Furthermore, the safety profile of these emerging treatments requires careful evaluation through well-designed longitudinal studies, assessing not only efficacy but also potential adverse effects, optimal dosing, and long-term impact on host physiology. Establishing clear safety guidelines and monitoring protocols will be essential for their successful clinical translation. Future research must focus on longitudinal human studies, mechanistic insights into microbe-host interactions, and the development of personalized microbiome-based interventions. Integrating microbiomics with other omics technologies could pave the way for a precision medicine approach in neurology. In conclusion, the GBA is not merely a bystander but an active player in neurodegenerative and neurotraumatic disease mechanisms. Leveraging its therapeutic potential could revolutionize current treatment strategies, offering novel, adjunctive solutions for complex and currently incurable conditions.

## Figures and Tables

**Table 4:** Studies reporting GBA in ALS.

Intervention	Key Findings—Related Targets and Pathways	Model/Limitations	Effect on ALS	Ref.
Administration of galactooligosaccharide (GOS) to SOD1G93A transgenic mice, a widely used animal model of ALS	GOS treatment extended survival, preserved spinal motor neurons, reduced neuroinflammation through decrease activation of microglia and astrocytes, and mitigated oxidative stress in SOD1G93A ALS mice	Mouse Model/Mouse model specificity, Gut–CNS link unclear	The findings suggest that GOS has neuroprotective effects in the SOD1G93A mouse model of ALS, potentially through its anti-inflammatory and antioxidant properties, leading to delayed disease progression and extended survival	[117]
Administration of Lacticaseibacillus rhamnosus HA-114 or its fatty acid extracts to *Caenorhabditis elegans* models of neurodegenerative diseases, including ALS HD	HA-114 supplementation reduced age-dependent paralysis and motor neuron degeneration in C. elegans models expressing mutant human proteins associated with ALS (FUS^S57Δ^ and TDP-43^A315T^) via fatty acid-mediated neuroprotection, requiring mitochondrial β-oxidation genes such as acdh-1 (ACADSB), kat-1 (ACAT1), and elo-6 (ELOVL3/6) and restoring neuronal lipid and energy homeostasis through a carnitine-independent mechanism	Caenorhabditis elegans models/non-mammalian model, Limited translational relevance, Simplified nervous system, No human data, Lack of Specialized Tissues in *C. elegans*	The study suggests that fatty acids produced by L. rhamnosus HA-114 can mitigate neurodegenerative phenotypes in *C. elegans* models by enhancing mitochondrial function and energy metabolism, highlighting a potential therapeutic avenue for age-related neurodegenerative diseases	[118]
Administration of the probiotic formulation AJ3 (Al-Pro) to assess its impact on immune cell activity in the context of ALS	AJ3 treatment reduced elevated IFN-γ levels secreted by natural killer (NK) and CD8⁺ T cells, contributing to immune-mediated damage in the central nervous system and peripheral organs and shifted the IFN-γ/IL-10 ratio toward an anti-inflammatory profile in ALS, outperforming other probiotic formulations in suppressing cytotoxic immune responses	ALS patient-derived samples And in vitro immune cell assays/Lacks in vivo validation, Human sample variability, Mechanism not fully explored, Translation to ALS uncertain, Patient heterogeneity considerations	The findings suggest that AJ3 may alleviate autoimmunity in ALS by modulating the activity of cytotoxic immune effectors, thereby potentially preventing motor neuron death and disease progression	[119]
Administration of butyrate (a short-chain fatty acid) or the probiotic formulation VSL#3 to TDP-43^A315T^ mutant mice, starting at 9 weeks of age	Treatment improved motor and gut function, restored barrier integrity by increase expression of tight junction proteins (ZO-1 and Claudin-5) and α-smooth muscle actin (α-SMA) in the colon, spinal cord, and brain, reduced mutant TDP-43 aggregation and glial activation, modulated gut microbiota toward butyrate production, and attenuated systemic inflammation by reducing serum levels of inflammatory cytokines (IL-6, IL-17, and IFN-γ) and LPS	Transgenic mice model/using a specific mouse model which limits the generalizability of the findings, lacking long-term follow-up, lacking human validation	The interventions delayed disease onset and progression in TDP-43 mutant mice by restoring gut and blood–brain barrier integrity, reducing neuroinflammation and protein aggregation, and modulating gut microbiota composition	[120]
Administration of 2% sodium butyrate (a natural bacterial product) in drinking water to G93A transgenic mice, a model for human ALS	Butyrate treatment in ALS models restored gut microbiota by increasing beneficial butyrate-producing bacteria, such as *Butyrivibrio fibrisolvens*, *Bacteroides*, *Odoribacter*, and *Eubacterium*, improved intestinal barrier integrity through upregulation of tight junction protein ZO-1, and normalized Paneth cell function by enhancing antimicrobial peptide-like lysozyme 1 and defensin 5 alpha expression. Additionally, it reduced the aggregation of mutant SOD1^G93A^ protein in both intestinal tissues and human epithelial cells, highlighting its therapeutic potential through gut–CNS axis modulation	Transgenic mice model/Single genetic mutation, Lack of mechanistic studies, Focused mainly on butyrate metabolite	Butyrate treatment in ALS models delayed disease onset, extended survival by an average of 38 days, and preserved neuromuscular function, indicating systemic benefits mediated through gut microbiota modulation and gut-CNS axis interaction	[121]
Administration of specific metabolites or compounds, including butyrate, lanthionine ketimine ethyl ester (LKE), and methionine sulfoximine (MSO), to ALS mouse models	Butyrate, LKE, and MSO each demonstrated therapeutic benefits in SOD1^G93A^ ALS mouse models by targeting distinct pathological mechanisms: butyrate reduced systemic inflammation by reducing serum levels of IL-17 and LPS, and restored gut-CNS communication; LKE promoted neuroprotection and functional recovery; and MSO mitigated excitotoxicity by lowering glutamate levels, thereby delaying disease onset and extending survival	Transgenic mice model/Animal model limitations, focus on specific metabolites, short treatment duration, small sample size	Administration of specific metabolites or compounds, including butyrate, lanthionine ketimine ethyl ester (LKE), and methionine sulfoximine (MSO), to ALS mouse models	[122]
Administration of sodium phenylbutyrate (SPB), a histone deacetylase (HDAC) inhibitor, to SOD1^G93A^ transgenic mice, a commonly used ALS model	SPB treatment in ALS mice significantly prolonged survival, improved motor function, and delayed disease onset. Mechanistically, SPB enhanced histone acetylation and upregulated anti-apoptotic genes (Bcl-2, Bcl-xL), indicating epigenetic regulation as a key mode of neuroprotection	Transgenic mice model/Mouse model may not fully replicate human ALS, the study did not assess long-term toxicity, adverse effects, Lack of dosing optimization.	The study demonstrates that SPB exerts neuroprotective effects in ALS by modulating epigenetic markers and gene expression, leading to delayed disease progression and extended survival in the mouse model	[123]
Combined administration of riluzole and sodium phenylbutyrate (NaPB) to SOD1^G93A^ transgenic mice, a commonly used ALS model	Combination therapy in ALS models extended survival by 21.5%, outperforming riluzole or NaPB alone. It improved neuropathology by reducing spinal cord atrophy, neuronal death, and astrogliosis. Mechanistically, it enhanced histone H4 acetylation and NF-κB p50 nuclear translocation, promoting anti-apoptotic gene expression through epigenetic modulation	Transgenic mice model/Focus on Specific Mechanism, clinical trials limitations, Long-term safety and efficacy requires further validation	The combined therapy demonstrated a synergistic neuroprotective effect, leading to prolonged survival, improved motor function, and reduced neurodegeneration in the ALS mouse model	[124]

## Data Availability

Not applicable.

## Abbreviations

Gut–brain axis	GBA
Central nervous system	CNS
Neurodegenerative diseases	NDDs
Short-chain fatty acids	SCFAs
Parkinson’s disease	PD
Alzheimer’s disease	AD
Amyotrophic lateral sclerosis	ALS
Huntington’s disease	HD
Traumatic brain injury	TBI
Multiple sclerosis	MS
Indoleamine 2,3-dioxygenase 1	IDO-1
Enteric nervous system	ENS
Fecal microbiota transplantation	FMT
Blood–brain barrier	BBB
Aryl hydrocarbon receptor	AhR
Lipopolysaccharides	LPS
Gut-associated lymphoid tissue	GALT
Beta-amyloid plaques	Aβ
Gamma-aminobutyric acid	GABA
Toll-like receptor 4	TLR4
Low-density lipoprotein	LDL
Parkinson Gut Prediction Method	PGPM
Non-steroidal anti-inflammatory drugs	NSAIDs
Glucagon-like peptide-1	GLP-1
Inflammatory bowel disease	IBD
Interferon-gamma	INF-γ
Interleukin-6	IL-6
Interleukin-10	IL-10
Interleukin-1β	IL-1β
Tumor necrosis factor-alpha	TNF-α
Transforming growth factor beta	TGF-β
Gastrointestinal	GI
Galactooligosaccharides	GOS
Fructo-oligosaccharides	FOS

## References

[1] Scalese G., Severi C. (2021). The pathophysiology of gut–brain connection. The Complex Interplay Between Gut-Brain, Gut-Liver, and Liver-Brain Axes.

[2] Arneth B.M. (2018). Gut-brain axis biochemical signalling from the gastrointestinal tract to the central nervous system: Gut dysbiosis and altered brain function. Postgrad. Med. J..

[3] Bhatt B., Patel K., Lee C.N., Moochhala S. (2024). The Microbial Blueprint: The Impact of Your Gut on Your Well-being.

[4] O’Riordan K.J., Collins M.K., Moloney G.M., Knox E.G., Aburto M.R., Fülling C., Morley S.J., Clarke G., Schellekens H., Cryan J.F. (2022). Short chain fatty acids: Microbial metabolites for gut-brain axis signalling. Mol. Cell. Endocrinol..

[5] Warren A., Nyavor Y., Zarabian N., Mahoney A., Frame L.A. (2024). The microbiota-gut-brain-immune interface in the pathogenesis of neuroinflammatory diseases: A narrative review of the emerging literature. Front. Immunol..

[6] Loh J.S., Mak W.Q., Tan L.K.S., Ng C.X., Chan H.H., Yeow S.H., Foo J.B., Ong Y.S., How C.W., Khaw K.Y. (2024). Microbiota–gut–brain axis and its therapeutic applications in neurodegenerative diseases. Signal Transduct. Target. Ther..

[7] Di Vincenzo F., Del Gaudio A., Petito V., Lopetuso L.R., Scaldaferri F. (2024). Gut microbiota, intestinal permeability, and systemic inflammation: A narrative review. Intern. Emerg. Med..

[8] Hanscom M., Loane D.J., Shea-Donohue T. (2021). Brain-gut axis dysfunction in the pathogenesis of traumatic brain injury. J. Clin. Invest..

[9] Yaqub M.O., Jain A., Joseph C.E., Edison L.K. (2025). Microbiome-Driven Therapeutics: From Gut Health to Precision Medicine. Gastrointest. Disord..

[10] Guo B., Zhang J., Zhang W., Chen F., Liu B. (2025). Gut microbiota-derived short chain fatty acids act as mediators of the gut–brain axis targeting age-related neurodegenerative disorders: A narrative review. Crit. Rev. Food Sci. Nutr..

[11] Cuesta C.M., Guerri C., Ureña J., Pascual M. (2021). Role of microbiota-derived extracellular vesicles in gut-brain communication. Int. J. Mol. Sci..

[12] Portincasa P., Bonfrate L., Vacca M., De Angelis M., Farella I., Lanza E., Khalil M., Wang D.Q.-H., Sperandio M., Di Ciaula A. (2022). Gut microbiota and short chain fatty acids: Implications in glucose homeostasis. Int. J. Mol. Sci..

[13] Ikeda T., Nishida A., Yamano M., Kimura I. (2022). Short-chain fatty acid receptors and gut microbiota as therapeutic targets in metabolic, immune, and neurological diseases. Pharmacol. Ther..

[14] La Rosa G., Lonardo M.S., Cacciapuoti N., Muscariello E., Guida B., Faraonio R., Santillo M., Damiano S. (2023). Dietary polyphenols, microbiome, and multiple sclerosis: From molecular anti-inflammatory and neuroprotective mechanisms to clinical evidence. Int. J. Mol. Sci..

[15] Fock E., Parnova R. (2023). Mechanisms of blood–brain barrier protection by microbiota-derived short-chain fatty acids. Cells.

[16] Zhou Y., Chen Y., He H., Peng M., Zeng M., Sun H. (2023). The role of the indoles in microbiota-gut-brain axis and potential therapeutic targets: A focus on human neurological and neuropsychiatric diseases. Neuropharmacology.

[17] Pickard J.M., Zeng M.Y., Caruso R., Núñez G. (2017). Gut microbiota: Role in pathogen colonization, immune responses, and inflammatory disease. Immunol. Rev..

[18] Yang J., Wise L., Fukuchi K.-I. (2020). TLR4 cross-talk with NLRP3 inflammasome and complement signaling pathways in Alzheimer’s disease. Front. Immunol..

[19] Majd S., Power J.H., Grantham H.J. (2015). Neuronal response in Alzheimer’s and Parkinson’s disease: The effect of toxic proteins on intracellular pathways. BMC Neurosci..

[20] Johansson-Lindbom B., Svensson M., Wurbel M.-A., Malissen B., Márquez G., Agace W. (2003). Selective generation of gut tropic T cells in gut-associated lymphoid tissue (GALT) requirement for GALT dendritic cells and adjuvant. J. Exp. Med..

[21] Su X., Yin X., Liu Y., Yan X., Zhang S., Wang X., Lin Z., Zhou X., Gao J., Wang Z. (2020). Gut Dysbiosis Contributes to the Imbalance of Treg and Th17 Cells in Graves’ Disease Patients by Propionic Acid. J. Clin. Endocrinol. Metab..

[22] Maciak K., Pietrasik S., Dziedzic A., Redlicka J., Saluk-Bijak J., Bijak M., Włodarczyk T., Miller E. (2021). Th17-related cytokines as potential discriminatory markers between neuromyelitis optica (Devic’s disease) and multiple sclerosis—A review. Int. J. Mol. Sci..

[23] Oemcke L.A., Anderson R.C., Altermann E., Roy N.C., McNabb W.C. (2021). The role of segmented filamentous bacteria in immune barrier maturation of the small intestine at weaning. Front. Nutr..

[24] Aljeradat B., Kumar D., Abdulmuizz S., Kundu M., Almealawy Y.F., Batarseh D.R., Atallah O., Ennabe M., Alsarafandi M., Alan A. (2024). Neuromodulation and the gut–brain axis: Therapeutic mechanisms and implications for gastrointestinal and neurological disorders. Pathophysiology.

[25] Jameson K.G., Kazmi S.A., Ohara T.E., Son C., Yu K.B., Mazdeyasnan D., Leshan E., Vuong H.E., Paramo J., Lopez-Romero A. (2025). Select microbial metabolites in the small intestinal lumen regulates vagal activity via receptor-mediated signaling. Iscience.

[26] Quadt L., Critchley H., Nagai Y. (2022). Cognition, emotion, and the central autonomic network. Auton. Neurosci..

[27] Bravo J.A., Forsythe P., Chew M.V., Escaravage E., Savignac H.M., Dinan T.G., Bienenstock J., Cryan J.F. (2011). Ingestion of Lactobacillus strain regulates emotional behavior and central GABA receptor expression in a mouse via the vagus nerve. Proc. Natl. Acad. Sci. USA.

[28] Van Autryve B. (2024). The Role of the Vagus Nerve in Neuroinflammation. Master’s Thesis.

[29] Kearns R. (2024). Gut–brain axis and neuroinflammation: The role of gut permeability and the kynurenine pathway in neurological disorders. Cell. Mol. Neurobiol..

[30] Zhao B., Yin Q., Fei Y., Zhu J., Qiu Y., Fang W., Li Y. (2022). Research progress of mechanisms for tight junction damage on blood–brain barrier inflammation. Arch. Physiol. Biochem..

[31] Munir M.U., Ali S.A., Chung K.H.K., Kakinen A., Javed I., Davis T.P. (2024). Reverse engineering the Gut-Brain Axis and microbiome-metabolomics for symbiotic/pathogenic balance in neurodegenerative diseases. Gut Microbes.

[32] Ardizzone A., Capra A.P., Repici A., Lanza M., Bova V., Palermo N., Paterniti I., Esposito E. (2024). Rebalancing NOX2/Nrf2 to limit inflammation and oxidative stress across gut-brain axis in migraine. Free Radic. Biol. Med..

[33] Casili G., Lanza M., Campolo M., Siracusa R., Paterniti I., Ardizzone A., Scuderi S.A., Cuzzocrea S., Esposito E. (2020). Synergic therapeutic potential of PEA-um treatment and NAAA enzyme silencing in the management of neuroinflammation. Int. J. Mol. Sci..

[34] DeTure M.A., Dickson D.W. (2019). The neuropathological diagnosis of Alzheimer’s disease. Mol. Neurodegener..

[35] Spillantini M.G., Goedert M. (2013). Tau pathology and neurodegeneration. Lancet Neurol..

[36] Gatz M., Reynolds C.A., Fratiglioni L., Johansson B., Mortimer J.A., Berg S., Fiske A., Pedersen N.L. (2006). Role of genes and environments for explaining Alzheimer disease. Arch. Gen. Psychiatry.

[37] Rabinovici G.D., Carrillo M.C., Forman M., DeSanti S., Miller D.S., Kozauer N., Petersen R.C., Randolph C., Knopman D.S., Smith E.E. (2017). Multiple comorbid neuropathologies in the setting of Alzheimer’s disease neuropathology and implications for drug development. Alzheimer’s Dement. Transl. Res. Clin. Interv..

[38] Rullmann M., Brendel M., Schroeter M.L., Saur D., Levin J., Perneczky R.G., Tiepolt S., Patt M., Mueller A., Villemagne V.L. (2022). Multicenter 18F-PI-2620 PET for in vivo Braak staging of tau pathology in Alzheimer’s disease. Biomolecules.

[39] Jack Jr C.R., Bennett D.A., Blennow K., Carrillo M.C., Dunn B., Haeberlein S.B., Holtzman D.M., Jagust W., Jessen F., Karlawish J. (2018). NIA-AA research framework: Toward a biological definition of Alzheimer’s disease. Alzheimer’s Dement..

[40] Pasupalak J.K., Rajput P., Gupta G.L. (2024). Gut microbiota and Alzheimer’s disease: Exploring natural product intervention and the Gut–Brain axis for therapeutic strategies. Eur. J. Pharmacol..

[41] Nakhal M.M., Yassin L.K., Alyaqoubi R., Saeed S., Alderei A., Alhammadi A., Alshehhi M., Almehairbi A., Al Houqani S., BaniYas S. (2024). The microbiota–gut–brain axis and neurological disorders: A comprehensive review. Life.

[42] Braniste V., Al-Asmakh M., Kowal C., Anuar F., Abbaspour A., Tóth M., Korecka A., Bakocevic N., Ng L.G., Kundu P. (2014). The gut microbiota influences blood-brain barrier permeability in mice. Sci. Transl. Med..

[43] Khan I.M., Nassar N., Chang H., Khan S., Cheng M., Wang Z., Xiang X. (2024). The microbiota: A key regulator of health, productivity, and reproductive success in mammals. Front. Microbiol..

[44] Kozhakhmetov S., Kaiyrlykyzy A., Jarmukhanov Z., Vinogradova E., Zholdasbekova G., Alzhanova D., Kunz J., Kushugulova A., Askarova S. (2024). Inflammatory Manifestations Associated with Gut Dysbiosis in Alzheimer’s Disease. Int. J. Alzheimer’s Dis..

[45] Quansah M., David M.A., Martins R., El-Omar E., Aliberti S.M., Capunzo M., Jensen S.O., Tayebi M. (2025). The Beneficial Effects of Lactobacillus Strains on Gut Microbiome in Alzheimer’s Disease: A Systematic Review. Healthcare.

[46] Łysiak K., Łysiak A. (2024). The role of the gut microbiome in Alzheimer’s disease. Prospect. Pharm. Sci..

[47] Kim Y., Lim J., Oh J. (2024). Taming neuroinflammation in Alzheimer’s disease: The protective role of phytochemicals through the gut− brain axis. Biomed. Pharmacother..

[48] Older E.A., Zhang J., Ferris Z.E., Xue D., Zhong Z., Mitchell M.K., Madden M., Wang Y., Chen H., Nagarkatti P. (2024). Biosynthetic enzyme analysis identifies a protective role for TLR4-acting gut microbial sulfonolipids in inflammatory bowel disease. Nat. Commun..

[49] Gu X., Fan M., Zhou Y., Zhang Y., Wang L., Gao W., Li T., Wang H., Si N., Wei X. (2024). Intestinal endogenous metabolites affect neuroinflammation in 5× FAD mice by mediating “gut-brain” axis and the intervention with Chinese Medicine. Alzheimer’s Res. Ther..

[50] Heston M.B., Hanslik K.L., Zarbock K.R., Harding S.J., Davenport-Sis N.J., Kerby R.L., Chin N., Sun Y., Hoeft A., Deming Y. (2023). Gut inflammation associated with age and Alzheimer’s disease pathology: A human cohort study. Sci. Rep..

[51] Yang J., Liang J., Hu N., He N., Liu B., Liu G., Qin Y. (2024). The gut microbiota modulates neuroinflammation in Alzheimer’s disease: Elucidating crucial factors and mechanistic underpinnings. CNS Neurosci. Ther..

[52] Solanki R., Karande A., Ranganathan P. (2023). Emerging role of gut microbiota dysbiosis in neuroinflammation and neurodegeneration. Front. Neurol..

[53] Saksida T., Koprivica I., Vujičić M., Stošić-Grujičić S., Perović M., Kanazir S., Stojanović I. (2017). Impaired IL-17 production in gut-residing immune cells of 5xFAD mice with Alzheimer’s disease pathology. J. Alzheimer’s Dis..

[54] Bemark M., Pitcher M.J., Dionisi C., Spencer J. (2024). Gut-associated lymphoid tissue: A microbiota-driven hub of B cell immunity. Trends Immunol..

[55] Vanderlip C.R., Stark C.E. (2024). APOE4 Increases Susceptibility to Amyloid, Accelerating Episodic Memory Decline. bioRxiv.

[56] Ciurleo G.C., de Azevedo O.G., Carvalho C.G., Vitek M.P., Warren C.A., Guerrant R.L., Oriá R.B. (2024). Apolipoprotein E4 and Alzheimer’s disease causality under adverse environments and potential intervention by senolytic nutrients. Clin. Nutr. ESPEN.

[57] Ardizzone A., Bova V., Casili G., Filippone A., Campolo M., Lanza M., Esposito E., Paterniti I. (2022). SUN11602, a bFGF mimetic, modulated neuroinflammation, apoptosis and calcium-binding proteins in an in vivo model of MPTP-induced nigrostriatal degeneration. J. Neuroinflamm..

[58] Berg D., Borghammer P., Fereshtehnejad S.-M., Heinzel S., Horsager J., Schaeffer E., Postuma R.B. (2021). Prodromal Parkinson disease subtypes—Key to understanding heterogeneity. Nat. Rev. Neurol..

[59] Borghammer P., Van Den Berge N. (2019). Brain-first versus gut-first Parkinson’s disease: A hypothesis. J. Park. Dis..

[60] Beekes M. (2021). The Neural Gut–Brain Axis of Pathological Protein Aggregation in Parkinson’s Disease and Its Counterpart in Peroral Prion Infections. Viruses.

[61] Hirayama M., Ohno K. (2021). Parkinson’s disease and gut microbiota. Ann. Nutr. Metab..

[62] Heravi F.S., Naseri K., Hu H. (2023). Gut microbiota composition in patients with neurodegenerative disorders (Parkinson’s and Alzheimer’s) and healthy controls: A systematic review. Nutrients.

[63] Heidari A., Yazdanpanah N., Rezaei N. (2022). The role of Toll-like receptors and neuroinflammation in Parkinson’s disease. J. Neuroinflamm..

[64] Kline E.M., Houser M.C., Herrick M.K., Seibler P., Klein C., West A., Tansey M.G. (2021). Genetic and environmental factors in P arkinson’s disease converge on immune function and inflammation. Mov. Disord..

[65] Liang X., Huang G., Wang Y., Andrikopoulos N., Tang H., Ding F., Li Y., Ke P.C. (2025). Polystyrene Nanoplastics Hitch-Hike the Gut–Brain Axis to Exacerbate Parkinson’s Pathology. ACS Nano.

[66] Ni Y., Tong Q., Xu M., Gu J., Ye H. (2025). Gut microbiota-induced modulation of the central nervous system function in Parkinson’s disease through the gut-brain Axis and short-chain fatty acids. Mol. Neurobiol..

[67] Leem Y.-H., Park J.-E., Park J.-S., Kim D.-Y., Park J.-M., Kim S.-E., Kang J.L., Kim H.-S. (2025). Activation of α7nAch receptors ameliorates α-synuclein pathology in the brain and gut of a subacute MPTP mouse model of Parkinson’s disease. Biomed. Pharmacother..

[68] Zhao Y., Lai Y., Darweesh S.K., Bloem B.R., Forsgren L., Hansen J., Katzke V.A., Masala G., Sieri S., Sacerdote C. (2024). Gut Microbial Metabolites and Future Risk of Parkinson’s Disease: A Metabolome-Wide Association Study. Mov. Disord..

[69] Wu J., Li C.-S., Huang W.-Y., Zhou S.-Y., Zhao L.-P., Li T., Li M.-A., Zhang M.-X., Qiao C.-M., Zhao W.-J. (2025). Gut microbiota promote the propagation of pathologic α-syn from gut to brain in a gut-originated mouse model of Parkinson’s disease. Brain Behav. Immun..

[70] Yu B., Zhang H., Zhang M. (2025). Deep learning-based differential gut flora for prediction of Parkinson’s. PLoS ONE.

[71] Wang W., Shi S., Su M., Li Y., Yao X., Jiang J., Yao W., Qin X., Wang Z., Tang C. (2025). Akkermansia muciniphila Akk11 Supplementation Attenuates MPTP-Induced Neurodegeneration by Inhibiting Microglial NLRP3 Inflammasome. Probiotics Antimicrob. Proteins.

[72] Qi Y., Dong Y., Chen J., Xie S., Ma X., Yu X., Yu Y., Wang Y. (2025). Lactiplantibacillus plantarum SG5 inhibits neuroinflammation in MPTP-induced PD mice through GLP-1/PGC-1α pathway. Exp. Neurol..

[73] Gao W., Wu X., Wang Y., Lu F., Liu F. (2024). Brazilin-Rich Extract from Caesalpinia sappan L. Attenuated the Motor Deficits and Neurodegeneration in MPTP/p-Induced Parkinson’s Disease Mice by Regulating Gut Microbiota and Inhibiting Inflammatory Responses. ACS Chem. Neurosci..

[74] Qiao C.-M., Ma X.-Y., Tan L.-L., Xia Y.-M., Li T., Wu J., Cui C., Zhao W.-J., Shen Y.-Q. (2025). Indoleamine 2, 3-dioxygenase 1 inhibition mediates the therapeutic effects in Parkinson’s disease mice by modulating inflammation and neurogenesis in a gut microbiota dependent manner. Exp. Neurol..

[75] Shao X., Wu T., Li M., Zheng M., Lin H., Qi X. (2025). Enterococcus faecalis Exerts Neuroprotective Effects via the Vagus Nerve in a Mouse Model of Parkinson’s Disease. Mol. Neurobiol..

[76] Shang M., Ning J., Zang C., Ma J., Yang Y., Jiang Y., Chen Q., Dong Y., Wang J., Li F. (2025). FLZ attenuates Parkinson’s disease pathological damage by increasing glycoursodeoxycholic acid production via down-regulating Clostridium innocuum. Acta Pharm. Sin. B.

[77] Hassan H.M., Abou-Hany H.O., Shata A., Hellal D., El-Baz A.M., ElSaid Z.H., Haleem A.A., Morsy N.E., Abozied R.M., Elbrolosy B.M. (2025). Vinpocetine and Lactobacillus Attenuated Rotenone-Induced Parkinson’s Disease and Restored Dopamine Synthesis in Rats through Modulation of Oxidative Stress, Neuroinflammation, and Lewy Bodies Inclusion. J. Neuroimmune Pharmacol..

[78] Chtioui N., Duval C., St-Pierre D.H. (2025). The impact of an active lifestyle on markers of intestinal inflammation in Parkinson’s disease: Preliminary findings. Clin. Park. Relat. Disord..

[79] Bolen M.L., Gomes B.N., Gill B., Menees K.B., Staley H., Jernigan J., McFarland N.R., Zimmermann E.M., Forsmark C.E., Tansey M.G. (2025). Peripheral blood immune cells from individuals with Parkinson’s disease or inflammatory bowel disease share deficits in iron storage and transport that are modulated by non-steroidal anti-inflammatory drugs. Neurobiol. Dis..

[80] Zaman V., Matzelle D., Banik N.L., Haque A. (2025). Dysregulation of Metabolic Peptides Precedes Hyperinsulinemia and Inflammation Following Exposure to Rotenone in Rats. Cells.

[81] Kempuraj D., Thangavel R., Natteru P., Selvakumar G., Saeed D., Zahoor H., Zaheer S., Iyer S., Zaheer A. (2016). Neuroinflammation induces neurodegeneration. J. Neurol. Neurosurg. Spine.

[82] Miner A.E., Dastgheyb N., Palomino M., Graves J.S. (2021). The Genetics of Multiple Sclerosis. Neuroimmunology: Multiple Sclerosis, Autoimmune Neurology and Related Diseases.

[83] Beard K., Srivastava S., Sharma K., Jaiswal S., Reddy S.P., Lisak R.P., Sriwastava S. (2024). Epidemiology, epigenetics, and etiological factors in multiple sclerosis. Clinical Aspects of Multiple Sclerosis Essentials and Current Updates.

[84] Garg N., Smith T.W. (2015). An update on immunopathogenesis, diagnosis, and treatment of multiple sclerosis. Brain Behav..

[85] Bjørklund G., Wallace D.R., Hangan T., Butnariu M., Gurgas L., Peana M. (2025). Cerebral iron accumulation in multiple sclerosis: Pathophysiology and therapeutic implications. Autoimmun. Rev..

[86] Falcão A.M., van Bruggen D., Marques S., Meijer M., Jäkel S., Agirre E., Samudyata n., Floriddia E.M., Vanichkina D.P., Ffrench-Constant C. (2018). Disease-specific oligodendrocyte lineage cells arise in multiple sclerosis. Nat. Med..

[87] Ghasemi N., Razavi S., Nikzad E. (2017). Multiple sclerosis: Pathogenesis, symptoms, diagnoses and cell-based therapy. Cell J. (Yakhteh).

[88] Zierfuss B., Larochelle C., Prat A. (2024). Blood–brain barrier dysfunction in multiple sclerosis: Causes, consequences, and potential effects of therapies. Lancet Neurol..

[89] Msheik A.N., Al Mokdad Z., Hamed F., Assi F., Jibbawi A., Saad J.-P., Mohanna R., Khoury A., Farhat M., Atat R. (2024). Epstein–Barr virus flare: A multiple sclerosis attack. Surg. Neurol. Int..

[90] Hedström A.K. (2023). Risk factors for multiple sclerosis in the context of Epstein-Barr virus infection. Front. Immunol..

[91] Bakhshi A., Eslami N., Norouzi N., Letafatkar N., Amini-Salehi E., Hassanipour S. (2024). The association between various viral infections and multiple sclerosis: An umbrella review on systematic review and meta-analysis. Rev. Med. Virol..

[92] Giordano A., Clarelli F., Pignolet B., Mascia E., Sorosina M., Misra K., Ferrè L., Bucciarelli F., Manouchehrinia A., Moiola L. (2025). Vitamin D affects the risk of disease activity in multiple sclerosis. J. Neurol. Neurosurg. Psychiatry.

[93] Wu J., Olsson T., Alfredsson L., Hedström A.K. (2024). Association between sun exposure habits and disease progression in multiple sclerosis. Eur. J. Neurol..

[94] Ouyang Q., Yu H., Xu L., Yu M., Zhang Y. (2024). Relationship between gut microbiota and multiple sclerosis: A scientometric visual analysis from 2010 to 2023. Front. Immunol..

[95] Yadav S.K., Ito N., Mindur J.E., Kumar H., Youssef M., Suresh S., Kulkarni R., Rosario Y., Balashov K.E., Dhib-Jalbut S. (2022). Fecal Lcn-2 level is a sensitive biological indicator for gut dysbiosis and intestinal inflammation in multiple sclerosis. Front. Immunol..

[96] Doenyas C., Clarke G., Cserjési R. (2025). Gut–brain axis and neuropsychiatric health: Recent advances. Sci. Rep..

[97] Cryan J.F., O’Riordan K.J., Cowan C.S., Sandhu K.V., Bastiaanssen T.F., Boehme M., Codagnone M.G., Cussotto S., Fulling C., Golubeva A.V. (2019). The microbiota-gut-brain axis. Physiol. Rev..

[98] Mostafavi Abdolmaleky H., Zhou J.-R. (2024). Gut microbiota dysbiosis, oxidative stress, inflammation, and epigenetic alterations in metabolic diseases. Antioxidants.

[99] Mowry E.M., Glenn J.D. (2018). The dynamics of the gut microbiome in multiple sclerosis in relation to disease. Neurol. Clin..

[100] Metaxouli K., Tsiou C., Dokoutsidou E., Margari N. (2025). Nutritional Intervention in Patients with Multiple Sclerosis, Correlation with Quality of Life and Disability—A Prospective and Quasi-Experimental Study. NeuroSci.

[101] Chu F., Shi M., Lang Y., Shen D., Jin T., Zhu J., Cui L. (2018). Gut microbiota in multiple sclerosis and experimental autoimmune encephalomyelitis: Current applications and future perspectives. Mediat. Inflamm..

[102] Al K.F., Craven L.J., Gibbons S., Parvathy S.N., Wing A.C., Graf C., Parham K.A., Kerfoot S.M., Wilcox H., Burton J.P. (2022). Fecal microbiota transplantation is safe and tolerable in patients with multiple sclerosis: A pilot randomized controlled trial. Mult. Scler. J. Exp. Transl. Clin..

[103] Dopkins N., Becker W., Miranda K., Walla M., Nagarkatti P., Nagarkatti M. (2021). Tryptamine attenuates experimental multiple sclerosis through activation of aryl hydrocarbon receptor. Front. Pharmacol..

[104] Melnikov M., Kasatkin D., Lopatina A., Spirin N., Boyko A., Pashenkov M. (2022). Serotonergic drug repurposing in multiple sclerosis: A new possibility for disease-modifying therapy. Front. Neurol..

[105] Muñoz-Jurado A., Escribano B.M., Caballero-Villarraso J., Galván A., Agüera E., Santamaría A., Túnez I. (2022). Melatonin and multiple sclerosis: Antioxidant, anti-inflammatory and immunomodulator mechanism of action. Inflammopharmacology.

[106] Shen C., Luo Z., Ma S., Yu C., Gao Q., Zhang M., Zhang H., Zhang J., Xu W., Yao J. (2022). Microbe-derived antioxidants reduce lipopolysaccharide-induced inflammatory responses by activating the Nrf2 pathway to inhibit the ROS/NLRP3/IL-1β signaling pathway. Int. J. Mol. Sci..

[107] Erny D., Hrabě de Angelis A.L., Jaitin D., Wieghofer P., Staszewski O., David E., Keren-Shaul H., Mahlakoiv T., Jakobshagen K., Buch T. (2015). Host microbiota constantly control maturation and function of microglia in the CNS. Nat. Neurosci..

[108] Yu H., Bai S., Hao Y., Guan Y. (2022). Fatty acids role in multiple sclerosis as “metabokines”. J. Neuroinflamm..

[109] Furusawa Y., Obata Y., Fukuda S., Endo T.A., Nakato G., Takahashi D., Nakanishi Y., Uetake C., Kato K., Kato T. (2013). Commensal microbe-derived butyrate induces the differentiation of colonic regulatory T cells. Nature.

[110] Pröbstel A.-K., Zhou X., Baumann R., Wischnewski S., Kutza M., Rojas O.L., Sellrie K., Bischof A., Kim K., Ramesh A. (2020). Gut microbiota–specific IgA+ B cells traffic to the CNS in active multiple sclerosis. Sci. Immunol..

[111] Dhasmana S., Dhasmana A., Narula A.S., Jaggi M., Yallapu M.M., Chauhan S.C. (2022). The panoramic view of amyotrophic lateral sclerosis: A fatal intricate neurological disorder. Life Sci..

[112] Van Es M.A., Hardiman O., Chio A., Al-Chalabi A., Pasterkamp R.J., Veldink J.H., Van den Berg L.H. (2017). Amyotrophic lateral sclerosis. Lancet.

[113] Ludolph A.C., Dietrich J., Dreyhaupt J., Kassubek J., Del Tredici K., Rosenbohm A. (2024). Clinical spreading of muscle weakness in amyotrophic lateral sclerosis (ALS): A study in 910 patients. J. Neurol..

[114] Xu Z., Xu R. (2024). Current potential diagnostic biomarkers of amyotrophic lateral sclerosis. Rev. Neurosci..

[115] Ciervo Y., Ning K., Jun X., Shaw P.J., Mead R.J. (2017). Advances, challenges and future directions for stem cell therapy in amyotrophic lateral sclerosis. Mol. Neurodegener..

[116] Al-Khayri J.M., Ravindran M., Banadka A., Vandana C.D., Priya K., Nagella P., Kukkemane K. (2024). Amyotrophic Lateral Sclerosis: Insights and New Prospects in Disease Pathophysiology, Biomarkers and Therapies. Pharmaceuticals.

[117] Song L., Gao Y., Zhang X., Le W. (2013). Galactooligosaccharide improves the animal survival and alleviates motor neuron death in SOD1G93A mouse model of amyotrophic lateral sclerosis. Neuroscience.

[118] Labarre A., Guitard E., Tossing G., Forest A., Bareke E., Labrecque M., Tétreault M., Ruiz M., Alex Parker J. (2022). Fatty acids derived from the probiotic Lacticaseibacillus rhamnosus HA-114 suppress age-dependent neurodegeneration. Commun. Biol..

[119] Chen P.-C., Kaur K., Ko M.-W., Huerta-Yepez S., Jain Y., Jewett A. (2023). Regulation of Cytotoxic Immune Effector Function by AJ3 Probiotic Bacteria in Amyotrophic Lateral Sclerosis (ALS). Crit. Rev. Immunol..

[120] Zhang Y., Xia Y., Sun J. (2024). Probiotics and microbial metabolites maintain barrier and neuromuscular functions and clean protein aggregation to delay disease progression in TDP43 mutation mice. Gut Microbes.

[121] Zhang Y.-g., Wu S., Yi J., Xia Y., Jin D., Zhou J., Sun J. (2017). Target intestinal microbiota to alleviate disease progression in amyotrophic lateral sclerosis. Clin. Ther..

[122] Ogbu D., Zhang Y., Claud K., Xia Y., Sun J. (2022). Target metabolites to slow down progression of amyotrophic lateral sclerosis in mice. Metabolites.

[123] Ryu H., Smith K., Camelo S.I., Carreras I., Lee J., Iglesias A.H., Dangond F., Cormier K.A., Cudkowicz M.E., Brown R.H. (2006). Sodium phenylbutyrate prolongs survival and regulates expression of anti-apoptotic genes in transgenic amyotrophic lateral sclerosis mice. J. Neurochem..

[124] Del Signore S.J., Amante D.J., Kim J., Stack E.C., Goodrich S., Cormier K., Smith K., Cudkowicz M.E., Ferrante R.J. (2009). Combined riluzole and sodium phenylbutyrate therapy in transgenic amyotrophic lateral sclerosis mice. Amyotroph. Lateral Scler..

[125] Yao J.-y., Liu T., Hu X.-r., Sheng H., Chen Z.-h., Zhao H.-y., Li X.-j., Wang Y., Hao L. (2024). An insight into allele-selective approaches to lowering mutant huntingtin protein for Huntington’s disease treatment. Biomed. Pharmacother..

[126] Landles C., Osborne G.F., Phillips J., Pico M.C., Nita I.M., Ali N., Greene J.R., Sathasivam K., Bates G.P. (2024). Mutant HTT protein decreases with CAG repeat expansion: Implications for therapeutics and bioassays. Brain Commun..

[127] Gil J.M., Rego A.C. (2008). Mechanisms of neurodegeneration in Huntington’s disease. Eur. J. Neurosci..

[128] Miguez A., Gomis C., Vila C., Monguió-Tortajada M., Fernández-García S., Bombau G., Galofré M., García-Bravo M., Sanders P., Fernández-Medina H. (2023). Soluble mutant huntingtin drives early human pathogenesis in Huntington’s disease. Cell. Mol. Life Sci..

[129] Dhingra H., Gaidhane S.A. (2023). Huntington’s disease: Understanding its novel drugs and treatments. Cureus.

[130] Pauli W.M., O’Reilly R.C., Yarkoni T., Wager T.D. (2016). Regional specialization within the human striatum for diverse psychological functions. Proc. Natl. Acad. Sci. USA.

[131] Aguirre C.G., Tshilenge K.-T., Battistoni E., Lopez-Ramirez A., Naphade S., Perez K., Song S., Mooney S.D., Melov S., Ehrlich M.E. (2023). Transcriptomic characterization reveals disrupted medium spiny neuron trajectories in Huntington’s disease and possible therapeutic avenues. bioRxiv.

[132] Dai Y., Wang H., Lian A., Li J., Zhao G., Hu S., Li B. (2023). A comprehensive perspective of Huntington’s disease and mitochondrial dysfunction. Mitochondrion.

[133] Cavalcante F.S.B.M., de Araújo L.L., Gomes G.F., Galvão S.L., Hipólito T.C.S.a.E., de Campos Piagentini M., dos Santos J.C.C. (2025). Role of Gut-Microbiome-Brain-Axis in Neurodegenerative Diseases: A Review on Mechanisms and Potential Therapeutics. Braz. J. Clin. Med. Rev..

[134] Wasser C.I., Mercieca E.-C., Kong G., Hannan A.J., McKeown S.J., Glikmann-Johnston Y., Stout J.C. (2020). Gut dysbiosis in Huntington’s disease: Associations among gut microbiota, cognitive performance and clinical outcomes. Brain Commun..

[135] Kong G., Lê Cao K.-A., Judd L.M., Li S., Renoir T., Hannan A.J. (2020). Microbiome profiling reveals gut dysbiosis in a transgenic mouse model of Huntington’s disease. Neurobiol. Dis..

[136] Adanyeguh I.M., Rinaldi D., Henry P.-G., Caillet S., Valabregue R., Durr A., Mochel F. (2015). Triheptanoin improves brain energy metabolism in patients with Huntington disease. Neurology.

[137] Kacher R., Mounier C., Caboche J., Betuing S. (2022). Altered cholesterol homeostasis in Huntington’s disease. Front. Aging Neurosci..

[138] Wasser C.I., Mercieca E.-C., Kong G., Hannan A.J., Allford B., McKeown S.J., Stout J.C., Glikmann-Johnston Y. (2023). A randomized controlled trial of probiotics targeting gut dysbiosis in Huntington’s disease. J. Huntington’s Dis..

[139] Gubert C., Choo J.M., Love C.J., Kodikara S., Masson B.A., Liew J.J., Wang Y., Kong G., Narayana V.K., Renoir T. (2022). Faecal microbiota transplant ameliorates gut dysbiosis and cognitive deficits in Huntington’s disease mice. Brain Commun..

[140] Pathak S., Nadar R., Kim S., Liu K., Govindarajulu M., Cook P., Watts Alexander C.S., Dhanasekaran M., Moore T. (2024). The influence of kynurenine metabolites on neurodegenerative pathologies. Int. J. Mol. Sci..

[141] Bondulich M.K., Fan Y., Song Y., Giorgini F., Bates G.P. (2021). Ablation of kynurenine 3-monooxygenase rescues plasma inflammatory cytokine levels in the R6/2 mouse model of Huntington’s disease. Sci. Rep..

[142] Rauchman S.H., Zubair A., Jacob B., Rauchman D., Pinkhasov A., Placantonakis D.G., Reiss A.B. (2023). Traumatic brain injury: Mechanisms, manifestations, and visual sequelae. Front. Neurosci..

[143] Cederberg D., Siesjö P. (2010). What has inflammation to do with traumatic brain injury?. Child’s Nerv. Syst..

[144] Rock K.L., Latz E., Ontiveros F., Kono H. (2009). The sterile inflammatory response. Annu. Rev. Immunol..

[145] Corps K.N., Roth T.L., McGavern D.B. (2015). Inflammation and neuroprotection in traumatic brain injury. JAMA Neurol..

[146] Karve I.P., Taylor J.M., Crack P.J. (2016). The contribution of astrocytes and microglia to traumatic brain injury. Br. J. Pharmacol..

[147] Stewart I.J., Howard J.T., Amuan M.E., Kennedy E., Balke J.E., Poltavskiy E., Walker L.E., Haigney M., Pugh M.J. (2024). Traumatic brain injury is associated with the subsequent risk of atrial fibrillation or atrial flutter. Heart Rhythm..

[148] Tsymbalyuk O., Gerzanich V., Simard J.M., Rathinam C.V. (2022). Traumatic brain injury alters dendritic cell differentiation and distribution in lymphoid and non-lymphoid organs. J. Neuroinflamm..

[149] Kerr N.A., de Rivero Vaccari J.P., Abbassi S., Kaur H., Zambrano R., Wu S., Dietrich W.D., Keane R.W. (2018). Traumatic brain injury-induced acute lung injury: Evidence for activation and inhibition of a neural-respiratory-inflammasome axis. J. Neurotrauma.

[150] Kharrazian D. (2015). Traumatic brain injury and the effect on the brain-gut axis. Altern. Ther. Health Med..

[151] Urban R.J., Pyles R.B., Stewart C.J., Ajami N., Randolph K.M., Durham W.J., Danesi C.P., Dillon E.L., Summons J.R., Singh C.K. (2020). Altered fecal microbiome years after traumatic brain injury. J. Neurotrauma.

[152] Godinho D.B., da Silva Fiorin F., Oliveira M.S., Furian A.F., Fighera M.R., Royes L.F.F. (2021). The immunological influence of physical exercise on TBI-induced pathophysiology: Crosstalk between the spleen, gut, and brain. Neurosci. Biobehav. Rev..

[153] Flinn H., Marshall A., Holcomb M., Cruz L., Soriano S., Treangen T.J., Villapol S. (2024). Antibiotic treatment induces microbiome dysbiosis and reduction of neuroinflammation following traumatic brain injury in mice. Res. Sq..

[154] Celorrio M., Abellanas M.A., Rhodes J., Goodwin V., Moritz J., Vadivelu S., Wang L., Rodgers R., Xiao S., Anabayan I. (2021). Gut microbial dysbiosis after traumatic brain injury modulates the immune response and impairs neurogenesis. Acta Neuropathol. Commun..

[155] Amaral W.Z., Kokroko N., Treangen T.J., Villapol S., Gomez-Pinilla F. (2024). Probiotic therapy modulates the brain-gut-liver microbiota axis in a mouse model of traumatic brain injury. Biochim. Biophys. Acta (BBA)-Mol. Basis Dis..

[156] Yanckello L.M., Fanelli B., McCulloch S., Xing X., Sun M., Hammond T.C., Colwell R., Gu Z., Ericsson A.C., Chang Y.-H. (2022). Inulin supplementation mitigates gut dysbiosis and brain impairment induced by mild traumatic brain injury during chronic phase. J. Cell. Immunol..

[157] Wu Y., Zhang J., Feng X., Jiao W. (2023). Omega-3 polyunsaturated fatty acids alleviate early brain injury after traumatic brain injury by inhibiting neuroinflammation and necroptosis. Transl. Neurosci..

[158] Bakkar N.-M.Z., Ibeh S., AlZaim I., El-Yazbi A.F., Kobeissy F. (2023). High-fat diets in traumatic brain injury: A ketogenic diet resolves what the Western diet messes up neuroinflammation and beyond. Diet and Nutrition in Neurological Disorders.

[159] Dong X., Su Y., Luo Z., Li C., Gao J., Han X., Yao S., Wu W., Tian L., Bai Y. (2024). Fecal microbiota transplantation alleviates cognitive impairment by improving gut microbiome composition and barrier function in male rats of traumatic brain injury following gas explosion. Front. Microbiol..

[160] Hu X., Jin H., Yuan S., Ye T., Chen Z., Kong Y., Liu J., Xu K., Sun J. (2023). Fecal microbiota transplantation inhibited neuroinflammation of traumatic brain injury in mice via regulating the gut–brain axis. Front. Cell. Infect. Microbiol..

[161] Du D., Tang W., Zhou C., Sun X., Wei Z., Zhong J., Huang Z. (2021). Fecal microbiota transplantation is a promising method to restore gut microbiota dysbiosis and relieve neurological deficits after traumatic brain injury. Oxidative Med. Cell. Longev..

[162] Davis IV B.T., Chen Z., Islam M.B., Timken M.E., Procissi D., Schwulst S.J. (2022). Fecal microbiota transfer attenuates gut dysbiosis and functional deficits after traumatic brain injury. Shock.

[163] Ardizzone A., Bova V., Casili G., Filippone A., Lanza M., Repici A., Esposito E., Paterniti I. (2023). bFGF-like Activity Supported Tissue Regeneration, Modulated Neuroinflammation, and Rebalanced Ca(2+) Homeostasis following Spinal Cord Injury. Int. J. Mol. Sci..

[164] Tang H., Gu Y., Jiang L., Zheng G., Pan Z., Jiang X. (2023). The role of immune cells and associated immunological factors in the immune response to spinal cord injury. Front. Immunol..

[165] Hasan A., Repici A., Capra A.P., Mannino D., Bova V., Catalfamo A., Campolo M., Paterniti I., Esposito E., Ardizzone A. (2025). CCR1 antagonist as a potential modulator of inflammatory, autophagic, and apoptotic markers in spinal cord injury. Neuropharmacology.

[166] Moura M.M., Monteiro A., Salgado A.J., Silva N.A., Monteiro S. (2024). Disrupted autonomic pathways in spinal cord injury: Implications for the immune regulation. Neurobiol. Dis..

[167] de la Cruz-Castillo E., García-Vences E. (2021). Beyond the Quality of Life in Bowel Dysfunction after Spinal Cord Injury: Approaches to the Consequences in Motility, Immune. Paraplegia.

[168] Mou Y., Du Y., Zhou L., Yue J., Hu X., Liu Y., Chen S., Lin X., Zhang G., Xiao H. (2022). Gut microbiota interact with the brain through systemic chronic inflammation: Implications on neuroinflammation, neurodegeneration, and aging. Front. Immunol..

[169] Chen X., Wang B., Al Mamun A., Du K., Wang S., Hu Q., Chen X., Lu Y., Du A., Wu Y. (2025). Pectin-Zein-IPA nanoparticles promote functional recovery and alleviate neuroinflammation after spinal cord injury. J. Nanobiotechnol..

[170] Wang J., Zhang N., Liu H.-Z., Wang J.-L., Zhang Y.-B., Su D.-D., Zhang L.-M., Li B.-D., Miao H.-T., Miao J. (2025). Hydrogen Sulfide (H_2_S) Generated in the Colon Induces Neuropathic Pain by Activating Spinal NMDA Receptors in a Rodent Model of Chronic Constriction Injury. Neurochem. Res..

[171] Hu X., Feng J., Lu J., Pang R., Zhang A., Liu J., Gou X., Bai X., Wang J., Chang C. (2024). Effects of exoskeleton-assisted walking on bowel function in motor-complete spinal cord injury patients: Involvement of the brain–gut axis, a pilot study. Front. Neurosci..

[172] Wu Z.-J., Zhao Y.-Y., Hao S.-J., Dong B.-B., Zheng Y.-X., Liu B., Li J. (2024). Combining fecal 16 S rRNA sequencing and spinal cord metabolomics analysis to explain the modulatory effect of PPARα on neuropathic pain. Brain Res. Bull..

[173] Yang X., Zhangyi Z., Yu A., Zhou Q., Xia A., Qiu J., Cai M., Chu X., Li L., Feng Z. (2024). GV-971 attenuates the progression of neuromyelitis optica in murine models and reverses alterations in gut microbiota and associated peripheral abnormalities. CNS Neurosci. Ther..

